# A Multi-Strategy Improved Zebra Optimization Algorithm for AGV Path Planning

**DOI:** 10.3390/biomimetics10100660

**Published:** 2025-10-01

**Authors:** Cunji Zhang, Chuangeng Chen, Jiaqi Lu, Xuan Jing, Wei Liu

**Affiliations:** 1 School of Mechanical and Electrical Engineering, Guilin University of Electronic Technology, Guilin 541004, China; zhangcunji@guet.edu.cn (C.Z.); 1955835175@mails.guet.edu.cn (C.C.); bingguoyouling@163.com (X.J.); 2 GUET-Nanning E-Tech Research Institute Co., Ltd., Nanning 530031, China; 3 Guangxi AGGF Home Furnishing Co., Ltd., Nanning 530200, China; aggf2003@163.com

**Keywords:** zebra optimization algorithm, multi-population search strategy, metropolis criterion, mutation operation, coati optimization algorithm, AGV path planning

## Abstract

The Zebra Optimization Algorithm (ZOA) is a metaheuristic algorithm inspired by the collective behavior of zebras in the wild. Like many other swarm intelligence algorithms, the ZOA faces several limitations, including slow convergence, susceptibility to local optima, and an imbalance between exploration and exploitation. To address these challenges, this paper proposes an improved version of the ZOA, termed the Multi-strategy Improved Zebra Optimization Algorithm (MIZOA). First, a multi-population search strategy is introduced to replace the traditional single population structure, dividing the population into multiple subpopulations to enhance diversity and improve global convergence. Second, the mutation operation of genetic algorithm (GA) is integrated with the Metropolis criterion to boost exploration capability in the early stages while maintaining strong exploitation performance in the later stages. Third, a novel selective aggregation strategy is proposed, incorporating the hunting behavior of the Coati Optimization Algorithm (COA) and Lévy flight to further enhance global exploration and convergence accuracy during the defense phase. Experimental evaluations are conducted on 23 benchmark functions, comparing the MIZOA with eight existing swarm intelligence algorithms. The performance is assessed using non-parametric statistical tests, including the Wilcoxon rank-sum test and the Friedman test. The results demonstrate that the MIZOA achieves superior global convergence accuracy and optimization performance, confirming its robustness and effectiveness. The MIZOA was evaluated on real-world engineering problems against seven algorithms to validate its practical performance. Furthermore, when applied to path planning tasks for Automated Guided Vehicles (AGVs), the MIZOA consistently identifies paths closer to the global optimum in both simple and complex environments, thereby further validating the effectiveness of the proposed improvements.

## 1. Introduction

An optimization problem is a mathematical formulation in which decision variables are adjusted to minimize or maximize an objective function subject to specific constraints. Traditional optimization methods, such as gradient descent and Newton’s method, rely heavily on gradient information and prior knowledge of the problem space, rendering them sensitive to initial conditions and often computationally expensive. As a result, they face significant limitations when applied to complex, high-dimensional, or non-differentiable problems. With the rapid advancement of artificial intelligence and deep learning, many real-world optimization tasks are increasingly characterized by incomplete feature space knowledge and dynamic environments. In this context, metaheuristic algorithms have attracted growing interest due to their strong global search capabilities and reduced reliance on problem-specific information [1]. These algorithms provide a promising alternative for solving complex optimization problems where traditional methods often fall short.

Metaheuristic algorithms are high-level heuristic optimization methods designed to obtain approximate optimal solutions in large-scale, complex, multimodal, and nonlinear problem spaces through intelligent search strategies. Compared with traditional deterministic optimization techniques, metaheuristics generally do not require gradient information or specific mathematical properties of the problem, thereby exhibiting strong global search capabilities and adaptability. These algorithms are particularly effective for addressing NP-hard problems and real-world engineering optimization tasks [2]. The fundamental principle of metaheuristic algorithms is to simulate natural mechanisms, biological behaviors, or physical phenomena to guide the search process, enabling them to efficiently escape local optima and gradually converge toward the global optimum. Based on their sources of inspiration, metaheuristic algorithms can be broadly categorized into four groups: (1) swarm intelligence algorithms inspired by collective behavior; (2) physics-based algorithms simulating natural processes; (3) human-inspired algorithms mimicking cognitive behaviors; and (4) evolutionary algorithms derived from biological evolution. Representative physics-based algorithms include Simulated Annealing (SA) [3], Gravitational Search Algorithm (GSA) [4], Thermal Exchange Optimization (TEO) [5], Black Hole Algorithm (BH) [6], and Electromagnetic Field Optimization (EFO) [7], among others. Human-inspired algorithms comprise the Brain Storm Optimization (BSO) [8], Gold Rush Optimization Algorithm (GRO) [9], Imperialist Competitive Algorithm (ICA) [10], and Teaching-Learning-Based Optimization (TLBO) [11], among others. Evolutionary algorithms encompass widely used approaches such as Genetic Algorithms (GA) [12], Differential Evolution (DE) [13], and Genetic Programming (GP) [14], among others.

Swarm intelligence algorithms are metaheuristic optimization methods inspired by the collective behavior of biological populations in nature. Over the past three decades, these algorithms have experienced significant development. Early approaches, such as Ant Colony Optimization (ACO) [15], Artificial Bee Colony (ABC) [16], and Particle Swarm Optimization (PSO) [17], laid the foundation for simulating cooperative and competitive behaviors observed in natural systems. More recent algorithms, including the Gray Wolf Optimizer (GWO) [18], Whale Optimization Algorithm (WOA) [19], Sparrow Search Algorithm (SSA) [20], Sand Cat Swarm Optimization (SCSO) [21], Black Eagle Optimizer (BEO) [22], Ant Lion Optimizer (ALO) [23], and others, have further expanded the available toolkit for addressing complex, nonlinear optimization problems. Owing to their strong robustness, parallel search capabilities, and minimal reliance on gradient information, swarm intelligence algorithms have demonstrated broad application potential across diverse fields, including engineering optimization, machine learning, data mining, and resource scheduling.

The Zebra Optimization Algorithm (ZOA) is a novel bio-inspired swarm intelligence algorithm proposed by Trojovská et al. in 2022 [24]. It is inspired by the foraging behavior and predator defense strategies exhibited by zebras in natural environments. As a population-based metaheuristic, ZOA models each zebra as an individual agent within the population, where each agent represents a candidate solution in the search space. Collectively, the population forms a diverse set of potential solutions. By simulating the social and movement behaviors of zebra herds, ZOA performs an efficient global search to locate the optimal solution within the problem space.

The ZOA has demonstrated broad applicability across various domains. For example, Elymany et al. [25] integrated ZOA into the design of hybrid renewable energy systems, combining it with maximum power point tracking techniques to optimize the power output of wind and solar energy sources, thereby enhancing overall system efficiency. Tong et al. [26] proposed a hybrid fault identification framework that integrates the Multi-Attraction Zebra Optimization Algorithm (MAZO) with LightGBM. In this approach, MAZO is employed to optimize the hyperparameters of the LightGBM classifier, thereby enhancing its capability to detect animal-induced intermittent faults in low-voltage distribution networks. The proposed method achieves high recognition accuracy while simultaneously reducing computational overhead and model complexity, making it well-suited for real-time monitoring applications. Yang et al. [27] proposed a hybrid model by integrating ZOA with a Backpropagation Neural Network (BPNN) to predict flow and heat transfer characteristics in manifold microchannels. They compared the performance of the ZOA-optimized BPNN with that of BPNNs tuned by Genetic Algorithm (GA), Particle Swarm Optimization (PSO), and Artificial Hummingbird Algorithm (AHA). Results demonstrated that the ZOA-BP model achieves superior generalization performance on unseen test data. To enhance early diagnosis of Autism Spectrum Disorder (ASD), Parvathy et al. [28] developed a hybrid deep learning architecture, ViT-ARDNet-LSTM, which leverages the Vision Transformer (ViT) for spatial feature extraction, Adaptive Residual DenseNets (ARDNet) for deep feature refinement, and LSTM for sequential pattern learning. A modified Zebra Optimization Algorithm (MZOA) was proposed to optimize the model’s hyperparameters and weights. Experimental evaluation demonstrates that the MZOA-optimized model achieves superior classification performance over conventional optimization techniques. Zhu et al. [29] developed a Dual-Branch Deep Convolutional Neural Network (DB-DCNN) model for lossless detection of concrete stress states using active wave signals. In their approach, the ZOA was applied during the adaptive thresholding stage of wavelet denoising, thereby enhancing the quality of the initial wave signals. Qiu et al. [30] developed a machine learning-based inversion model for estimating soil alkali-hydrolyzable nitrogen (SAN) content using satellite remote sensing data across different bare soil periods. The ZOA was employed to optimize the hyperparameters of the model (e.g., number of trees, learning rate, and maximum depth in XGBoost). Experimental results demonstrated that the ZOA-optimized model achieved superior prediction accuracy compared to models tuned by other metaheuristic algorithms, with improvements in R^2^ and RMSE on independent test datasets. Lv et al. [31] proposed an Improved Zebra Optimization Algorithm (IZOA) to optimize an attention-enhanced Gated Recurrent Unit (GRU) model for predicting continuous knee joint angle changes in lower-limb exoskeleton robots using surface electromyography (sEMG) signals. The IZOA incorporates Tent and Logistic chaotic mappings to enhance population diversity during initialization, along with a memory-based learning strategy to improve convergence stability. The performance of the IZOA-optimized GRU model was evaluated against models tuned by Genetic Algorithm (GA), standard ZOA, Liver Cancer Algorithm (LCA), and Pied Kingfisher Optimizer (PKO). Results demonstrated that the IZOA-based approach achieves superior prediction accuracy and faster convergence, validating its effectiveness in parameter optimization for sEMG-driven motion prediction. Integrated Sensing and Communication (ISAC) is a promising enabling technology for sixth-generation (6G) networks. Sun et al. [32] proposed a semi-grant-free non-orthogonal random access (SGF-NORA) scheme to enhance throughput and connectivity in ISAC networks under massive machine-type communication device (MTCD) scenarios. To address the access control subproblem, the ZOA was employed to optimize user scheduling and resource allocation. Extensive simulation results demonstrated that the SGF-NORA scheme significantly improves network throughput and access success rate, validating its effectiveness for large-scale MTCD deployments. Wei et al. [33] proposed an Energy-Efficient Emergency Air–Ground Integrated Heterogeneous AIoT (E3-HetAIoT) architecture to enhance energy management in smart cities. The framework integrates a cell-based clustering mechanism, where a multi-objective Zebra Optimization Algorithm (M-ZOA) is employed to jointly optimize the selection of cluster heads (CHs) and super nodes (UNs). The optimization process aims to minimize energy consumption, balance load distribution, and maximize network lifetime. Simulation results showed that the M-ZOA-based approach significantly reduces energy usage and extends network longevity compared with baseline methods, validating its effectiveness for large-scale AIoT deployments.

Since ZOA is a relatively recent addition to the family of swarm intelligence algorithms, most existing studies have focused on direct applications of the original algorithm, while relatively few have explored its methodological enhancements. According to the No Free Lunch Theorem (NFL), no single optimization algorithm is universally effective for all problem types [34]. An algorithm that excels in one domain may encounter significant limitations in others. Similarly, ZOA faces certain challenges, including slow convergence speed, susceptibility to premature convergence, and an imbalance between exploration and exploitation, particularly when addressing complex or high-dimensional problems. For instance, when applied to Automated Guided Vehicle (AGV) path planning [35], ZOA may suffer from premature convergence and fail to identify the globally optimal shortest path. In summary, the ZOA and its improved variants have found broad applications in diverse domains, including communications, power systems, healthcare, robotics, the Internet of Things, and agriculture, demonstrating significant potential as meta-heuristic optimization methods. Among these, machine learning represents a particularly prominent application area, where ZOA-based approaches are extensively used for hyperparameter optimization in neural networks and other models.

To address the aforementioned limitations, this paper proposes a Multi-Strategy Improved Zebra Optimization Algorithm (MIZOA). The proposed algorithm incorporates four enhancement strategies into the original ZOA framework: a multi-population search mechanism, mutation operation inspired by GA, the Metropolis criterion [36], and a selective aggregation strategy. (1) The multi-population search strategy partitions the initial population into several subpopulations, thereby reducing the risk of premature convergence and enhancing the algorithm’s capability to escape local optima. (2) Mutation operation derived from genetic algorithms are introduced to increase population diversity and assist the algorithm in avoiding stagnation in local optima during the search process. (3) The Metropolis criterion allows for the probabilistic acceptance of inferior solutions, which helps preserve population diversity and improves global convergence performance. (4) The selective aggregation strategy is designed to balance exploration and exploitation by integrating the hunting behavior of the Coati Optimization Algorithm (COA) [37] with Lévy flight characteristics [38,39], thereby strengthening the algorithm’s ability to tackle complex, high-dimensional optimization problems. In the experimental phase, the convergence behavior, stability, and overall optimization performance of MIZOA were evaluated using a comprehensive set of benchmark functions. To rigorously assess its effectiveness, statistical analyses were conducted on the obtained results. Seven well-established metaheuristic algorithms were selected for comparative evaluation: the original ZOA, GWO, WOA, Chameleon Swarm Algorithm (CSA) [40], Firefly Algorithm (FA) [41], Sine–Cosine Algorithm (SCA) [42], and Aquila Optimizer (AO) [43]. Finally, MIZOA was applied to the AGV path planning problem, and comparative experiments were conducted against six other algorithms in both simple and complex environmental maps to evaluate its performance in solving two-dimensional grid-based path planning tasks.

The main contributions of this study are summarized as follows:

- An improved variant of ZOA, termed MIZOA, is proposed by integrating multiple enhancement strategies to improve the algorithm’s overall performance.
- A multi-population search strategy based on K-means clustering is introduced into the traditional ZOA framework, effectively enhancing population diversity and reducing the risk of premature convergence caused by a single pioneer individual.
- A GA-inspired mutation operation is incorporated into the foraging phase, introducing additional randomness and promoting the exploration of unvisited regions in the solution space, thereby improving the algorithm’s global search capability.
- The Metropolis criterion is integrated into the mutation process, allowing suboptimal solutions to be accepted with a certain probability. This mechanism enhances the algorithm’s ability to escape local optima and improves its convergence performance on complex multimodal problems
- A selective aggregation strategy combining Lévy flight and the position update mechanism of COA is proposed for the defense phase, which balances global exploration and local exploitation, further enhancing the robustness of the search process.
- Performance evaluations of MIZOA and the comparative algorithms are conducted on a comprehensive set of benchmark functions, followed by statistical analyses using the Wilcoxon rank-sum test and the Friedman test to assess the significance of performance differences. To comprehensively evaluate the efficiency and practicality of MIZOA, runtime comparisons were conducted among the eight algorithms. Furthermore, all algorithms were applied to a set of constrained real-world engineering optimization problems to assess their performance in practical scenarios.
- The proposed MIZOA is applied to AGV path planning problems in grid-based workshop environments. Comparative experiments with several representative algorithms validate the superior convergence speed, optimization accuracy, and stability of MIZOA in both simple and complex environments.

The remainder of this paper is organized as follows: Section 2 outlines the fundamental principles of ZOA, providing essential background on its search mechanism. Section 3 presents the proposed MIZOA, detailing the four enhancement strategies integrated into the original framework, along with their theoretical foundations and implementation procedures. Section 4 conducts a series of comparative experiments between MIZOA and seven widely recognized metaheuristic optimization algorithms to evaluate the effectiveness of the proposed improvements. Statistical analyses, including the Wilcoxon rank-sum test and Friedman test, are performed to ensure the robustness and significance of the results. In Section 5, the MIZOA is applied to AGV system path planning problems to assess its capability in generating optimal paths within two-dimensional grid-based environments. Section 6 concludes the paper by summarizing the key contributions, discussing the limitations of the current work, and suggesting potential directions for future research to facilitate further developments in this field.

## 2. Zebra Optimization Algorithm (ZOA)

In this study, the optimization problem under consideration can be generally formulated as follows:

(1) $$\underset{X\in R^{n}}{\min}f(X)$$

(2) $$g_{i}(X)\leq 0,\;i=1,2,\ldots ,m,$$

(3) $$h_{j}(X)=0,\;j=1,2,\ldots ,p,$$

(4) $$X_{L}\leq X\leq X_{U},$$

In this context, $X={\left(x_{1},x_{2},\ldots ,x_{d}\right)}^{T}$ is a d-dimensional decision variable vector, *f*(*x*) is the objective function, $g_{i}(X)$ and $h_{j}(X)$ denote the inequality and equality constraint functions, respectively, and $X_{L}$ and $X_{U}$ represent the lower and upper bounds of the decision variables. The algorithm operates on a population $P=\{X^{\left(1\right)},\ldots ,X^{\left(N\right)}\}\subset S$, iteratively updating its members to minimize *f* (the objective function) while satisfying all constraints.

This problem formulation covers both unconstrained and constrained optimization scenarios. For unconstrained problems, the constraint conditions are omitted. In many real-world applications, the search space is complex, and the objective function may be non-differentiable and non-convex. Under such conditions, traditional deterministic mathematical programming methods can be inefficient or prone to being trapped in local optima. Therefore, this study adopts a swarm intelligence–based metaheuristic approach—the Zebra Optimization Algorithm (ZOA) and its improved variants—to approximate solutions to the proposed optimization model, aiming to balance global search capability and convergence efficiency.

The ZOA draws inspiration from the group behavior of zebras on the African savannah [24]. In their natural habitat, zebra herds typically forage together and employ various defensive strategies to ward off predator attacks. Researchers have abstracted this pattern of cooperative and competitive behavior into ZOA, which is designed to find global optimal solutions to optimization problems. Zebras are herbivores, and their daily activities mainly consist of two phases. During the foraging phase, zebras move within their habitat in search of food. This movement is not random but is guided by pioneer zebra within the herd. This behavioral characteristic guides the algorithm’s initial search within the solution space, accelerating the process of locating the global optimal solution or a position close to it. During the defense phase, when faced with threats from predators, zebras adopt different strategies for survival. For example, they may gather together to form a stronger group that confuses predators or quickly disperse to increase individual chances of survival. These strategies are incorporated into the algorithm’s secondary search process, enhancing its flexibility and helping it escape local optima.

### 2.1. Initialization

The algorithm begins by randomly generating a population of zebra individuals, where the position of each individual represents a potential solution to the optimization problem. Collectively, the population constitutes a set of candidate solutions. Depending on the dimensionality of the problem, each zebra’s position is represented by a vector with the corresponding number of dimensions. The initialization process of the zebra population is defined by Equation (5).

(5) $$X={\left[\begin{array}{c} X_{1}\\ \vdots \\ X_{i}\\ \vdots \\ X_{N} \end{array}\right]}_{N\times m}={\left[\begin{array}{ccccc} x_{1,1} & \ldots & x_{1,j} & \ldots & x_{1,m}\\ \vdots & \ddots & \vdots & \ddots & \vdots \\ x_{i,1} & \ldots & x_{i,j} & \ldots & x_{i,m}\\ \vdots & \ddots & \vdots & \ddots & \vdots \\ x_{N,1} & \ldots & x_{N,j} & \ldots & x_{N,m} \end{array}\right]}_{N\times m}$$

Here, *X* denotes the position matrix of the entire zebra population. where *N* is the number of individuals in the population and *m* is the dimensionality of the optimization problem. Each row of the matrix represents the position vector of a single individual, while each column contains the positional components of all individuals along a specific dimension.

The quality of each individual position within the zebra population is evaluated using a fitness function. In practical optimization problems, a smaller fitness value typically indicates a better solution, while a larger value corresponds to a poorer one. The fitness of the entire zebra population is calculated according to Equation (6).

(6) $$F={\left[\begin{array}{c} F_{1}\\ \vdots \\ F_{i}\\ \vdots \\ F_{N} \end{array}\right]}_{N\times 1}={\left[\begin{array}{c} F(X_{1})\\ \vdots \\ F(X_{i})\\ \vdots \\ F(X_{N}) \end{array}\right]}_{N\times 1}$$

In this equation, *F* denotes the fitness value vector of the entire population. where *F_i_* represents the fitness value of the *i*th individual in the zebra population. *N* is the total number of individuals, and *F*(*X*) denotes the fitness function used to evaluate each solution.

### 2.2. Foraging

During the foraging phase, the zebra population follows the pioneer zebra, defined as the individual with the best position in the population—that is, the zebra with the lowest fitness value. The remaining individuals update their positions by moving toward the pioneer zebra, which accelerates convergence and enables the algorithm to approach the global optimum more efficiently. The corresponding position update mechanism is formulated in Equations (7) and (8).

(7) $$x_{i,j}^{new,P1}=x_{i,j}+r\cdot (PZ_{j}-I\cdot x_{i,j})$$

(8) $$X_{i}=\left\{\begin{array}{ll} X_{i}^{new,P1}, & F_{i}^{new,P1}\leq F_{i};\\ X_{i}, & \mathrm{else}. \end{array}\right.$$

where $x_{i,j}^{new,P1}$ represents the updated position of the *i*th zebra in the *j*th dimension during the foraging phase (denoted as *P*1). *X_i_* denotes the original position of the *i*th zebra in the *j*th dimension. *r* is a random number uniformly distributed in the range [0, 1], and *PZ*_j_ indicates the positional component of the pioneer zebra in the *j*th dimension. $F_{i}^{new,P1}$ is the fitness value of the *i*th zebra at its new position during the foraging phase, and *F_i_* is its fitness value at the original position. Equation (4) specifies that a new position is accepted according to the greedy selection principle. That is, the new position is accepted only if its fitness value is lower than that of the current position.

### 2.3. Defense

The predators encountered by zebra populations can be classified into two categories, assuming equal probability of encountering each type. The first category includes predators with strong individual predatory capabilities, such as lions. The second category consists of predators with relatively weaker individual predatory abilities, such as hyenas. In response to these different types of threats, zebra populations adopt distinct defensive strategies. *S*_1_ denotes the dispersal escape strategy, which is employed when facing highly aggressive predators like lions. *S*_2_ represents the group aggregation strategy, typically used when confronting less aggressive predators such as hyenas. The acceptance criteria for new positions during the defense phase continue to follow the rules of the greedy algorithm. Based on the above principles, the corresponding mathematical models are presented in Equations (9) and (10).

(9) $$x_{i,j}^{new,P2}=\left\{\begin{array}{ll} S_{1}:x_{i,j}+R\cdot (2r-1)\cdot \left(1-\frac{t}{T}\right)\cdot x_{i,j}, & P_{s}\leq 0.5;\\ S_{2}:x_{i,j}+r\cdot (AZ_{j}-I\cdot x_{i,j}), & \mathrm{else}, \end{array}\right.$$

(10) $$X_{i}=\left\{\begin{array}{ll} X_{i}^{new,P2}, & F_{i}^{new,P2}<F_{i};\\ X_{i}, & \mathrm{else}, \end{array}\right.$$

where $x_{i,j}^{new,P2}$ represents the new position of the *i*th zebra in the *j*th dimension during the defense stage. R=0.01. *t* denotes the current iteration number. *T* is the maximum number of iterations. $P_{s}$ is a random number uniformly distributed in [0, 1]. *R* is a constant parameter set to 0.01. When $P_{s}\leq 0.5$, strategy $S_{1}$ is executed, otherwise strategy $S_{2}$ is applied. $AZ_{j}$ represents the position component in the *j*th dimension of an attacked zebra, which is randomly selected from the population. $F_{i}^{new,P2}$ denotes the fitness value corresponding to the new position of the *i*th zebra in the defense phase.

### 2.4. ZOA Pseudocode

The pseudocode of the ZOA algorithm is presented in Algorithm 1. It outlines the iterative process by which the zebra population updates their positions to search for the global optimum, simulating the social foraging behavior of zebras in the wild.
**Algorithm 1:** ZOA pseudocode**Input**: Fitness function (objective function) F(x), population size N, maximum number of iterations T, problem dimensionality *m* Initialize the zebra population **for** *t* = 1:T Update pioneer zebra position (*PZ*) **for** *i* = 1:N  **P1: Foraging phase**  Calculate the new position of the *i*th zebra using Equation (7)  Evaluate the new position of the *i*th zebra using Equation (8)  **P2: Defense phase**  $P_{S}$ = *rand*  **if** $P_{S}\leq 0.5$   Calculate the new position of the *i*th zebra using the *S*_1_ strategy in Equation (9)  **else**   Calculate the new position of the *i*th zebra using the *S*_2_ strategy in Equation (9)  **end if**  Evaluate the new position of the *i*th zebra using Equation (10) **end for** Record the best solution obtained so far, along with its corresponding fitness value **end for** **Output**: The optimal solution and its corresponding fitness value

## 3. MIZOA

This section introduces the MIZOA. First, the conventional single population framework is modified by dividing the zebra population into multiple subpopulations, with each subpopulation independently exploring the solution space. This approach helps prevent premature convergence and reduces the risk of getting trapped in local optima. Second, mutation operation from GA is integrated into the zebra foraging phase, while the Metropolis criterion is adopted as the acceptance rule for new positions. These enhancements improve population diversity and strengthen the algorithm’s global convergence capability. Finally, a selective aggregation strategy is incorporated during the population’s defense phase. This strategy integrates the position update mechanism of COA with Lévy flight, simulating the behavior of a raccoon attacking prey in a tree. The use of Lévy flight further balances the exploration and exploitation abilities of the algorithm. Collectively, these improvements enhance the algorithm’s overall convergence accuracy, convergence speed, and solution stability.

These modifications preserve the biological inspiration underlying the original ZOA while addressing its limitations in tackling complex optimization problems. Through these targeted improvements, MIZOA demonstrates superior performance in terms of both solution quality and computational efficiency, as confirmed by extensive experimental results presented in the subsequent sections.

### 3.1. Multi-Population Search Strategies

The traditional ZOA employs a single population in which all individuals follow the movement of a single pioneer zebra (PZ) during the foraging phase. In multimodal optimization problems, if the direction chosen by the pioneer zebra deviates from the global optimum, the entire population may be misled, leading to premature convergence at a local optimum from which it is difficult to escape. To address this limitation, the proposed multi-population search strategy [44] adopts a clustering-based method to divide the original population into multiple subpopulations. Considering both computational efficiency and overhead, the K-means clustering algorithm is employed in this study due to its simplicity and low computational complexity [45]. According to Arthur and Vassilvitskii (2007), the standard k-means procedure, which involves alternating between assignment steps and centroid update steps, is not only straightforward to implement but also tends to converge rapidly. This combination of ease of use and speed contributes to its popularity as a clustering technique, even though its performance can be significantly affected by the initial placement of centroids [46].

**Figure 1:**
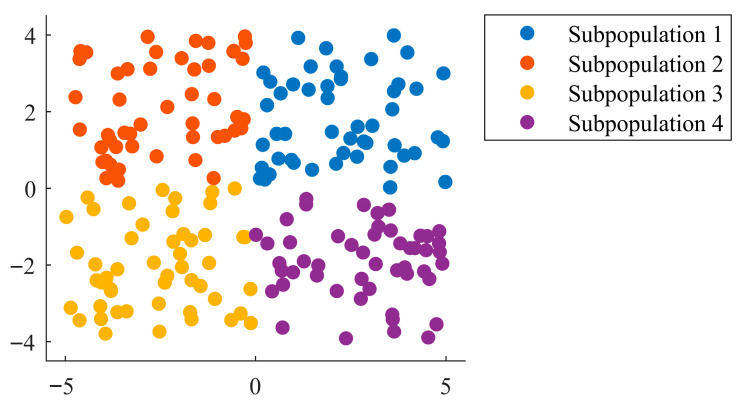
Schematic diagram of multi-population search strategy.

Each subpopulation independently explores the solution space and maintains its own sub-pioneer zebra, fulfilling a role equivalent to the global pioneer in the original algorithm. During the foraging phase, individuals within each subpopulation update their positions by following their respective sub-pioneer zebra. This strategy substantially reduces the risk of the entire population becoming trapped in a local optimum due to the influence of a single leader. Moreover, it distributes the search effort across multiple regions of the solution space, thereby enhancing population diversity and improving the overall robustness of the algorithm. The corresponding mathematical model is presented in Equation (11). A schematic illustration of the proposed multi-population search strategy is presented in Figure 1. In this example, the population is divided into four subpopulations to enhance diversity and improve the overall search capability.

(11) $$PZ_{sub}(sub=1,2\ldots k)$$

where *PZ_i_* denotes the sub-pioneer zebra of the *i*th subpopulation, and *k* represents the total number of subpopulations.

### 3.2. Mutation Operation and Metropolis Criteria

Mutation is a fundamental operator in GA, designed to simulate the phenomenon of gene variation in biological evolution [47]. By randomly altering certain genes within individuals, mutation introduces new genetic material into the population, allowing the algorithm to explore previously unexplored regions of the solution space. This mechanism enhances the algorithm’s ability to escape local optima and increases the probability of converging toward the global optimum [48]. In the original ZOA, the foraging phase relies exclusively on the position update mechanism defined in Equation (3). However, this mechanism alone may be insufficient to maintain adequate population diversity throughout the optimization process. To this end, a GA mutation operator is introduced into this phase, specifically employing a time-adaptive uniform mutation strategy. During the early stages of the optimization process, the focus is on global exploration to maintain population diversity, while in the later stages, the emphasis shifts toward local exploitation to accelerate convergence [49]. Each individual in the population is assigned a predefined probability of undergoing mutation. This strategy is consistent with the principles of genetic variation in biological evolution, contributing to increased population diversity and preventing excessive aggregation and duplication of individual positions within the population. Additionally, the constant coefficient *r* originally employed in Equation (3) is replaced with a time-adaptive coefficient to further improve the algorithm’s convergence speed and overall performance. The revised mathematical formulation is provided in Equation (12).

(12) $$x_{sub,i,j}^{new,P1}=\left\{\begin{array}{ll} S_{1}:x_{sub,i,j}+{\left(1-\frac{t}{T}\right)}^{a}\cdot (PZ_{sub,j}-I\cdot x_{sub,i,j}), & P_{f}>0.1\\ S_{2}:x_{sub,i,j}+0.2\cdot (ub_{j}-lb_{j})\cdot (1-\frac{t}{T}), & else \end{array}\right.$$

where $x_{sub,i,j}^{new,P1}$ denotes the new position of the *i*th zebra in the *j*th dimension within the subpopulation. *a* is a control parameter, set to 0.01 in this study, I∈{1,2}. *ub_j_* denotes the upper bound of the *j*th dimension in the solution space, while *lb_j_* denotes the corresponding lower bound. And *P_f_* is a decision variable, where $P_{f}>0.1$, Strategy 1 (*S*_1_) is executed, otherwise, Strategy 2 (*S*_2_) is applied.

The Metropolis criterion is an acceptance-rejection strategy commonly used in Monte Carlo methods [36], particularly in Markov Chain Monte Carlo (MCMC) techniques. It provides a probabilistic mechanism for determining whether to accept a new state based on the current state, even when the energy or cost of the new state is higher. This mechanism enables the system to accept inferior solutions with a certain probability, thereby enhancing population diversity and reducing the likelihood of premature convergence [50]. As a core component of both Monte Carlo methods and SA algorithms, the Metropolis criterion facilitates the probabilistic acceptance of suboptimal solutions. This feature helps the algorithm escape from local optima and improves its global search capability. The mathematical formulations of the Metropolis criterion are given in Equations (13) and (14).

(13) $$P=\left\{\begin{array}{ll} 1, & \;\Delta E\leq 0\\ \exp\left(-\frac{\Delta E}{k_{B}\cdot T_{temp}}\right), & \;\Delta E>0 \end{array}\right.$$

(14) $$\Delta E=E^{new}-E^{old}$$

where ΔE denotes the energy difference between the current and new system states. P is the acceptance probability of the system. $T_{temp}$ represents the system temperature, and $k_{B}$ is the Boltzmann constant. When ΔE≤0, the system directly accepts the new state. Otherwise, the new state is accepted with a certain probability determined by the Metropolis criterion.

Based on the Metropolis criterion described above, the acceptance rule for new positions generated through the zebra individual mutation operation (*S*_2_) is defined by Equations (15)–(18). For the *S*_1_ strategy, the acceptance criterion remains consistent with that of the original ZOA and is specified in Equation (19).

(15) $$\Delta F_{i}=\frac{F_{i}^{new}-F_{i}^{old}}{F_{sub}^{\max}-F_{sub}^{\min}}$$

(16) $$T_{temp}=T_{0}\cdot (1-\frac{t}{T})$$

(17) $$P=\left\{\begin{array}{ll} 1, & \;\Delta F_{i}\leq 0\\ \exp\left(-\frac{\Delta F_{i}}{k_{B}\cdot T_{temp}}\right), & \;\Delta F_{i}>0 \end{array}\right.$$

(18) $$X_{sub,i}=\left\{\begin{array}{ll} X_{sub,i}^{new,P1}, & \;r\leq P;\\ X_{sub,i}, & \mathrm{else}. \end{array}\right.$$

(19) $$X_{sub,i}=\left\{\begin{array}{ll} X_{sub,i}^{new,P1}, & F_{i}^{new,P1}<F_{i};\\ X_{sub,i}, & \mathrm{else}, \end{array}\right.$$

where $\Delta F_{i}$ denotes the normalized fitness difference in the *i*th zebra. $F_{sub}^{\max}$ and $F_{sub}^{\min}$ represent the maximum and minimum fitness values within the subpopulation, respectively. $T_{0}$ is the initial system temperature, and $T_{temp}$ is the real-time system temperature. *r* is a uniformly distributed random number within the interval [0, 1]. In this study, $k_{B}$ is set to 1. P denotes the acceptance probability for the new position of a zebra individual. When P=1, the new position is directly accepted. when P<1, the new position is accepted with probability P.

### 3.3. Selective Aggregation Strategy

During the defense phase of the ZOA, there is a 50% probability that the zebra population will aggregate around the attacked zebra (AZ). While this aggregation behavior enhances local exploitation, it can reduce population diversity and compromise convergence accuracy. To overcome this limitation, a selective aggregation strategy is introduced, which incorporates the position update mechanism of COA combined with Lévy flight. This strategy effectively enhances population diversity while maintaining local search precision, thereby further improving the balance between exploration and exploitation during the defense phase [51].

Lévy flight is a random walk strategy characterized by a heavy-tailed probability distribution [38,39], in which step lengths follow a Lévy distribution, as defined in Equation (20). This behavior is commonly observed in nature, including the foraging patterns of albatrosses, the pollen-searching behavior of bees, and the flight trajectories of flies. The distinguishing feature of Lévy flight lies in its combination of frequent short steps with occasional long-distance moves. The step length generation mechanism is provided in Equation (21). This property allows Lévy flight to perform fine-grained local searches through short steps, while the intermittent long jumps increase the probability of escaping local optima and exploring distant regions of the search space. In contrast to the normal distribution, which exhibits light tails, the Lévy distribution assigns a higher probability to larger step lengths, enabling a broader and more diversified search range than traditional random walks [52]. When integrated into the defense phase of ZOA, Lévy flight further improves the balance between local exploitation and global exploration. This enhancement strengthens the algorithm’s adaptability and search performance, particularly in complex, high-dimensional, and multimodal optimization problems [53].

(20) $$L(s)\sim \frac{\mu }{s^{1+\beta }},\;0<\beta <2$$

where μ is the scale parameter. β is the stability parameter that controls the heaviness of the distribution’s tail, and S denotes the step size.

(21) $$s=\frac{u}{|v{|}^{1/\beta }}$$

where $u\sim N(0,\sigma_{u}^{2})$, $v\sim N(0,\sigma_{v}^{2})$, $\sigma_{v}=1$, and $\sigma_{u}$ are shown in Equation (22).

(22) $$\sigma_{u}={\left(\frac{\Gamma (1+\beta )\cdot \sin(\pi \beta /2)}{\Gamma ((1+\beta )/2)\cdot \beta \cdot 2^{(\beta -1)/2}}\right)}^{1/\beta }$$

The COA is a swarm intelligence algorithm proposed by Dehghani et al. [37] in 2023. It is inspired by the cooperative hunting and predator evasion behaviors exhibited by coatis in their natural habitat. During the hunting phase, the population is divided into two equal groups: one half climbs trees to flush out prey, while the other remains on the ground to ambush the escaping prey. When the prey is driven from the tree and falls to the ground, its landing position is randomly determined. The ground-based coatis then update their search positions based on this random location, thereby completing the hunting process. The mathematical model representing this cooperative hunting behavior is formulated in Equation (23).

(23) $$x_{i,j}=\left\{\begin{array}{ll} x_{i,j}+r\cdot (Iguana_{j}^{G}-I\cdot x_{i,j}), & F_{Iguana^{G}}<F_{i}\\ x_{i,j}+r\cdot (x_{i,j}-Iguana_{j}^{G}), & F_{Iguana^{G}}\geq F_{i} \end{array}\right.$$

where $x_{i,j}$ represents the position of the *i*th coati in the *j*th dimension. $Iguana_{j}^{G}$ denotes the random position of the prey on the ground in the *j*th dimension, I∈{1,2}, r∈[0,1]. $F_{Iguana^{G}}$ represents the fitness value of the prey, and $F_{i}$ is the fitness value of the *i*th coati.

Based on the above principles, a selective aggregation strategy is proposed to enhance the defense phase of ZOA. The improved position update mechanism for the defense phase is presented in Equations (24) and (25). The acceptance criteria for evaluating new positions remain based on the greedy selection mechanism, as defined in Equation (26).

(24) $$x_{sub,i,j}^{new,P2}=\left\{\begin{array}{ll} S_{3}:x_{sub,i,j}+R\cdot (2r-1)\cdot {\left(1-\frac{t}{T}\right)}^{b}\cdot x_{sub,i,j}, & P_{s}\leq 0.5;\\ x_{sub,i,j}^{COA}, & \mathrm{else}, \end{array}\right.$$

(25) $$x_{sub,i,j}^{COA}=\left\{\begin{array}{ll} S_{4}:x_{sub,i,j}+r\cdot s\cdot (AZ_{sub,j}-I\cdot x_{sub,i,j}), & F_{AZ_{sub}}\leq F_{sub,i}\\ S_{5}:x_{sub,i,j}+r\cdot s\cdot (x_{sub,i,j}-AZ_{sub,j}), & F_{AZ_{sub}}>F_{sub,i} \end{array}\right.$$

(26) $$X_{sub,i}=\left\{\begin{array}{ll} X_{sub,i}^{new,P2}, & F_{i}^{new,P2}<F_{i};\\ X_{sub,i}, & \mathrm{else}, \end{array}\right.$$

where *R* is set to 0.01, *b* is a control parameter. *P_s_* is a uniformly distributed random number within the interval [0, 1]. $AZ_{sub,j}$ denotes the position of the *j*th dimension of the attacked zebra in the *sub*th subpopulation, and *s* is the step size determined by the Lévy flight mechanism. When $P_{s}\leq 0.5$, the $S_{3}$ strategy is executed. Otherwise, the COA strategy is applied. Furthermore, if $F_{AZ_{sub}}<F_{sub,i}$, the $S_{4}$ strategy is performed; Otherwise, the $S_{5}$ strategy is executed.

The selective aggregation strategy enhances the algorithm’s generalization capability by replacing the traditional single aggregation behavior with a more adaptive mechanism. This strategy integrates the cooperative hunting behavior of COA and the Lévy flight mechanism, enabling more effective exploration of the search space. For instance, when solving unimodal functions, the strategy can accelerate convergence speed and improve accuracy. In the case of multimodal functions, it helps maintain population diversity and increases the likelihood of converging to the global optimum.

### 3.4. MIZOA’s Pseudocode and Flowchart

**Figure 2:**
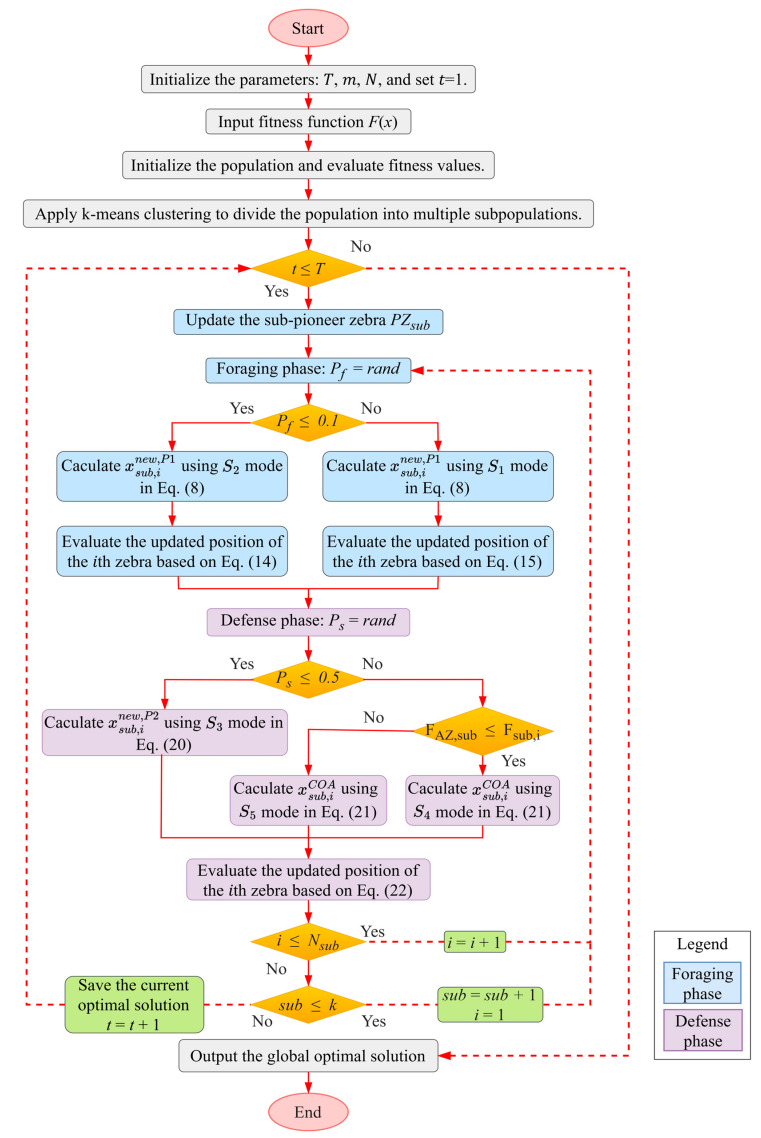
Flowchart of MIZOA.

The MIZOA begins by initializing the input parameters, after which a multi-population search strategy is applied using K-means clustering to divide the initial single population into several subpopulations. The algorithm then proceeds to the foraging phase, where each individual has a probability of 10% to undergo mutation; otherwise, the position is updated using the original ZOA position update mechanism. In the subsequent defense phase, the selective aggregation strategy is executed with a probability of 50%, which is consistent with the probability of employing the S3 strategy. These two phases are iteratively performed until the specified maximum number of iterations is reached. Finally, the algorithm outputs the global optimal solution. The overall flowchart of the MIZOA is presented in Figure 2. The pseudocode of MIZOA is summarized in Algorithm 2, which outlines the overall structure and key computational steps of the algorithm, including initialization, mutation, selective aggregation, and position update mechanisms.
**Algorithm 2:** MIZOA pseudocode**Input**: Fitness function (objective function) F(x), population size N, maximum number of iterations T, problem dimensionality *m*, Number of subpopulations *k* Initialize the zebra population K-means clustering divides the population **for** *t* = 1:T Update sub-pioneer zebra position ($PZ_{sub}$) **for**
sub=1:k  **for** *i* = $1:N_{sub}$   **P1: Foraging phase**   $P_{f}=rand$   **if** $P_{f}\leq 0.1$    Calculate the new position of the *i*th zebra using the *S_2_* strategy in Equation (12)    Evaluate the new position of the *i*th zebra using Equation (18)   **else**    Calculate the new position of the *i*th zebra using the *S_1_* strategy in Equation (12)    Evaluate the new position of the *i*th zebra using Equation (19)   **end if**   **P2: Defense phase**   $P_{S}$ = rand   **if** $P_{S}\leq 0.5$    Calculate the new position of the *i*th zebra using the *S_3_* strategy in Equation (24)   **else**    **if** $F_{AZ,sub}\leq F_{sub,i}$     Calculate the new position of the *i*th zebra using the *S_4_* strategy in Equation (25)    **else**     Calculate the new position of the *i*th zebra using the *S_5_* strategy in Equation (26)    **end if**   **end if**   Evaluate the new position of the *i*th zebra using Equation (26)  **end for** **end for** Record the best solution obtained so far, along with its corresponding fitness value **end for** **Output**: The optimal solution and its corresponding fitness value

### 3.5. Computational Complexity

The time complexity of the initialization phase in the ZOA is O(*N* × *m*), where *N* denotes the population size and *m* is the problem dimension. The foraging phase has a time complexity of O(*T* × *N* × *m*), with *T* representing the maximum number of iterations. The defense phase shares the same computational complexity as the foraging phase. Therefore, the overall time complexity of ZOA is O((*2* × *T* + *1*) × *N* × *m*), which can be asymptotically simplified to O(*T* × *N* × *m*).

For MIZOA, the variation operations in the foraging phase, the Metropolis criterion, and the selective aggregation strategy in the defense phase do not significantly increase the algorithm’s time complexity. The time complexity of the initialization phase remains O(*N* × *m*), while that of both the foraging and defense phases is O(*T* × *N* × *m*). However, the introduction of a multi-population search strategy based on the K-means clustering algorithm introduces an additional computational cost of O(*N* × *m* × *k* × *Tₖ*), where *k* denotes the number of subpopulations and *Tₖ* is the maximum number of iterations for the K-means algorithm. Consequently, the overall time complexity of MIZOA becomes O(*N* × *m* × (*2* × *T* + *1* + *k* × *Tₖ*)), which asymptotically simplifies to O(*T* × *N* × *m*). Therefore, the computational complexity of MIZOA remains comparable to that of the original ZOA and is of the same order of magnitude.

### 3.6. Algorithm Convergence Analysis

Reference [54] presents a theoretical framework for analyzing the convergence of random search algorithms. Given a function *f* from *R^n^* to *R* and *S* a subset of *R^n^*. We seek a point *x* in *S* which minimizes *f* on *S* or at least which yields an acceptable approximation of the infimum of *f* on *S*. The convergence of a random search algorithm can be rigorously proven under the following two conditions.

**Condition** **1.** f(D(x,ξ))≤f(x) and if ξ∈S, f(D(x,ξ))≤f(ξ)

*where D is a mapping; x denotes the current solution vector; f is the fitness function; ξ is a vector in Rⁿ representing the random perturbation applied to x; and S denotes the solution space.*

(27) $$F_{best-so-far}=\left\{\begin{array}{ll} F_{current}, & F_{current}<F_{best-so-far}\\ F_{best-so-far}, & \mathrm{else} \end{array}\right.$$

In MIZOA, the best-so-far solution is updated at the end of each iteration according to a greedy selection strategy, as defined in Equation (27). Specifically, the update occurs only if the best fitness value in the current population is lower than the previously recorded best value. Consequently, the sequence of best fitness values is non-increasing (i.e., monotonically decreasing for strict improvements), which is clearly reflected in the convergence curve.

**Condition** **2.** *For any (Borel) subset A of S with v(A) > 0, we have that*
(28) $$\prod_{k=0}^{\infty }\left[1-\mu_{k}(A)\right]=0$$

The $\mu_{k}$ are probability measures corresponding to distribution functions defined on $R^{n}$. In most cases, *v*(*A*) refers to the n*n*-dimensional volume of the set *A*; more generally, it denotes the Lebesgue measure of *A*. The quantity 1 − *μk*(*A*) is the probability that the set *A* is not hit at iteration *k*.

In MIZOA, the state evolution follows a Markov process, where *x*(*t* + 1) depends only on *x*(*t*). As *t*→∞, the probability of sampling from a neighborhood *A*⊂*S* of the optimal solution converges to one, satisfying the convergence condition stated in Equation (28).

Under Condition 1 and Condition 2, MIZOA is provably convergent to the global optimum, ensuring its global convergence property.

## 4. Experiments and Analysis of Results

To comprehensively evaluate the performance of the proposed MIZOA, a set of benchmark test functions is employed in this section. The evaluation primarily focuses on its convergence behavior and stability. Convergence performance is assessed by analyzing the speed and accuracy with which MIZOA approaches the optimal solution under a predefined maximum number of iterations, compared with other existing algorithms. Algorithmic stability is examined through multiple independent runs, with the mean and standard deviation of the obtained results calculated to quantify its robustness across different trials. Furthermore, nonparametric statistical tests, including the Wilcoxon rank-sum test and the Friedman test, are conducted to provide rigorous statistical validation of the algorithm’s performance.

### 4.1. Experimental Environment and Parameter Settings

All experiments in this study were conducted on a personal computer equipped with an Intel^®^ Core™ i7-8700 CPU @ 3.2 GHz and 16.0 GB of RAM (Lenovo, Beijing, China). To maintain consistency in the computational environment, all algorithms were implemented using MATLAB R2023b. For a fair and unbiased comparison, the population size *N* was uniformly set to 30, the problem dimension *m* was fixed at 30, and the maximum number of iterations *T* was set to 500 for all algorithms. Each algorithm was independently executed 30 times on each benchmark function, and the mean and standard deviation of the results were recorded to evaluate performance in terms of convergence accuracy and stability.

In this study, a total of 23 benchmark test functions are employed to comprehensively evaluate the performance of the proposed algorithm [55]. These benchmark functions are widely adopted in the literature to examine various algorithmic characteristics, including convergence speed, robustness, global exploration capability, and local exploitation efficiency. Specifically, functions F1~F7 are unimodal test functions without local optima, making them well-suited for assessing the convergence speed and local search ability of the algorithm. Functions F8~F13 are multimodal test functions containing multiple local optima, with the number of local minima increasing alongside problem dimensionality. These functions are particularly valuable for evaluating the global exploration capability and the algorithm’s ability to avoid premature convergence. The test functions are configured with 30 dimensions, a commonly adopted dimensionality in benchmarking optimization algorithms, to ensure sufficient complexity while maintaining reasonable computational cost. Functions F14~F23 are multimodal functions with fixed dimensionality; although defined in lower-dimensional spaces, their complex and rugged landscapes present greater optimization challenges. For unimodal functions, convergence speed is considered the primary performance metric due to the presence of a unique global optimum. In contrast, for multimodal functions, the final optimization result serves as the key evaluation indicator, as it reflects the algorithm’s global search ability. Detailed definitions and characteristics of all benchmark functions are presented in Table 1, Table 2 and Table 3.

The comparative algorithms selected in this study include the ZOA, Gray Wolf Optimizer (GWO), Whale Optimization Algorithm (WOA), Chameleon Swarm Algorithm (CSA), Firefly Algorithm (FA), Sine Cosine Algorithm (SCA), and Aquila Optimizer (AO). The parameter settings for each algorithm are listed in Table 4 and follow the standard configurations recommended in their respective original publications to ensure fairness and consistency in comparative evaluation.

### 4.2. Sensitivity Analysis of Key Parameters

This section presents a sensitivity analysis of the MIZOA, examining how different parameter combinations affect its performance. The key parameters under investigation are the mutation probability and the number of subpopulations. An appropriate mutation probability enhances population diversity, facilitating convergence to the global optimum. Multi-population search strategies partition the population into several subpopulations, which improves exploration and helps the algorithm escape local optima. However, an excessively large or small number of subpopulations may impair performance by disrupting the balance between exploration and exploitation. The mutation probability is set to 0.05, 0.1, and 0.2, while the number of subpopulations k takes values of 3, 5, and 7. The resulting parameter combinations are summarized in Table 5.

A total of nine parameter combinations are evaluated. Table 6 presents the experimental results across 23 benchmark functions. The population size is fixed at 30, and each combination is tested over 30 independent runs, with a maximum of 500 iterations per run. To assess both performance and stability, the mean and standard deviation of the final objective function values are calculated.

As shown in Table 6, the experimental results across the nine parameter settings exhibit minimal variation for most benchmark functions. The majority of the results fall within the same order of magnitude, indicating that the MIZOA is robust to parameter changes and demonstrates low sensitivity. For the unimodal functions (F1–F7), only F5 shows notable performance variation, with the best result achieved under Scenario 6. In the case of multimodal functions (F8–F13), the mean performance remains stable across most scenarios, with only F12 and F13 exhibiting slight fluctuations—yet still within the same order of magnitude. Standard deviations show minor differences, suggesting relatively consistent performance. For the fixed-dimensional multimodal test functions (F14–F23), MIZOA achieves convergence to the global optimum in almost all scenarios. However, variations in standard deviation are observed for certain functions under different parameter settings. For instance, in the case of function F23, the standard deviation varies across parameter combinations, indicating differences in algorithmic stability. Nevertheless, these variations remain within an acceptable range. Therefore, a mutation probability of 0.1 and a subpopulation number of k = 5 are selected as the final parameter settings for this study. Based on the above analysis, MIZOA demonstrates low sensitivity to parameter variations and strong robustness in most cases, making it reliable and easy to implement.

### 4.3. Convergence Performance Evaluation

**Figure 3:**
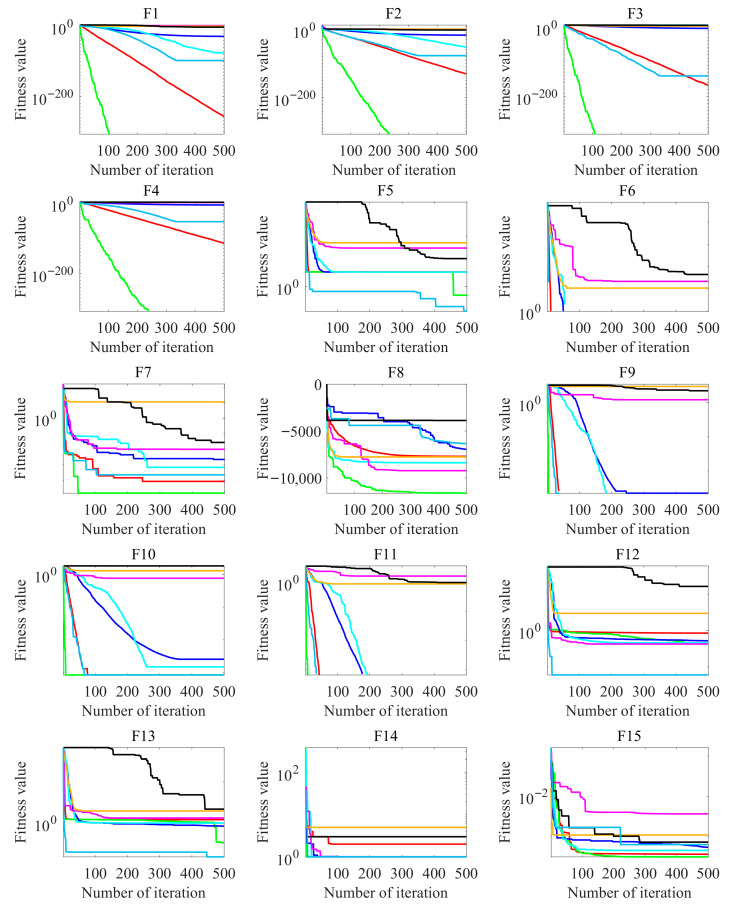

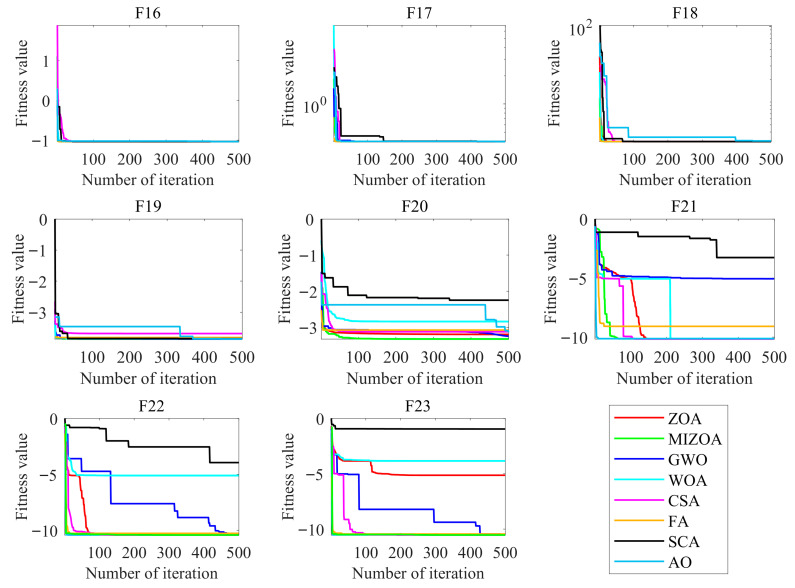
Comparison of the convergence curves of the algorithms on benchmark functions.

Figure 3 presents the convergence curve comparisons of eight optimization algorithms across 23 benchmark test functions. In the experiment, the population size was set to 30, the maximum number of iterations was limited to 500, and each algorithm was independently executed 30 times. The average fitness value was computed over these runs, and the convergence curves were plotted based on the averaged results. The slope of each curve reflects the convergence speed—steeper slopes indicate faster convergence. For unimodal test functions (F1~F7), MIZOA demonstrates significant advantages, maintaining superior performance in both convergence speed and accuracy across six of the seven functions, except for F5. This strongly validates the effectiveness of its multi-strategy improvements and highlights the robustness of the algorithm. Notably, in optimizing the *F6* function, which exhibits a step-like characteristic, MIZOA achieves convergence within a single iteration, showcasing its exceptional instantaneous optimization capability. Although MIZOA’s convergence speed and accuracy are slightly outperformed by the AO algorithm on F5, it still delivers a substantial improvement over the traditional ZOA. Specifically, MIZOA improves convergence accuracy by approximately two orders of magnitude compared to ZOA on F5.

In the experiments conducted on the multimodal test functions (F8~F13), MIZOA consistently demonstrates superior performance in both convergence speed and solution accuracy on five out of six functions. Although MIZOA does not achieve the highest final accuracy on F11, where WOA obtains better results, it maintains the fastest convergence rate among all compared algorithms on this function. For F12, while the convergence speed of MIZOA is slightly lower than that of AO, it achieves a substantial improvement in solution accuracy compared to the original ZOA, ranking second only to AO across all tested algorithms. These results further validate the effectiveness of the proposed multi-strategy modifications in enhancing both the global search capability and convergence stability of the algorithm in complex multimodal environments.

In the experiments involving fixed-dimensional multimodal test functions (F14~F23), MIZOA exhibits competitive convergence performance, achieving a favorable balance between convergence speed and solution accuracy. Notably, on functions F21, F22, and F23, MIZOA outperforms the traditional ZOA, demonstrating that the proposed improvements effectively preserve strong convergence capabilities even in complex, low-dimensional problem landscapes. These results further confirm the adaptability and robustness of MIZOA across diverse optimization scenarios.

The experimental results presented in Figure 3 indicate that the multi-strategy improved MIZOA consistently achieves convergence performance within the top 20% across all 23 benchmark test functions. Compared to the traditional ZOA, MIZOA demonstrates substantial improvements in both convergence speed and solution accuracy. These results effectively validate the effectiveness of the proposed hybrid multi-strategy improvement framework, which not only enhances the algorithm’s global exploration capability but also accelerates the convergence process.

### 4.4. Stability Analysis

Table 7 presents the stability test results for eight optimization algorithms across 23 benchmark functions. The evaluation metrics include the mean (AVG) and standard deviation (STD) obtained from 30 independent runs for each function. As shown in the table, for unimodal test functions (F1~F7), MIZOA achieves the best performance on all functions except F5, consistently yielding the lowest mean and standard deviation among the compared algorithms. This result indicates that the proposed enhancements effectively improve both the robustness and convergence accuracy of the algorithm. For function F5, although MIZOA’s stability is slightly inferior to that of the AO algorithm, it still outperforms the traditional ZOA by approximately one order of magnitude and ranks second overall. This outcome highlights a significant performance gain relative to the baseline algorithm. Regarding multimodal test functions (F8~F13), MIZOA obtains the best results on all functions except F12 and F13. Notably, on F8, the mean value improves by an order of magnitude, reflecting a marked enhancement in the algorithm’s global search capability. The integration of multi-population search strategies and mutation operation plays a crucial role in achieving these improvements. Although MIZOA does not attain the best results on F12 and F13, it still shows substantial improvements over the traditional ZOA in both mean and standard deviation, further confirming the effectiveness of the proposed enhancement strategies. For fixed-dimensional multimodal test functions (F14~F23), MIZOA achieves optimal performance on all functions except F14, demonstrating its strong capability in handling complex and rugged landscapes. Compared to ZOA, MIZOA exhibits significant improvements in disturbance resistance and escaping local optima, achieving a well-balanced trade-off between solution accuracy and algorithmic stability. These findings further validate the contributions of key mechanisms such as selective aggregation and the Metropolis acceptance criterion.

To further evaluate the performance of MIZOA, a recently proposed improved variant, MZOA, was included in the comparative analysis (see Table 7). For the unimodal test functions (F1–F7), both algorithms achieve identical results on the first four functions. However, MIZOA obtains solutions closer to the global optimum on F5 and F6, indicating better convergence accuracy. For the multimodal functions (F8–F13), MZOA outperforms MIZOA only on F12, while the results for F9 and F11 are identical between the two algorithms. These findings suggest that MIZOA’s multi-strategy framework is more effective, demonstrating stronger global search capability. Regarding the fixed-dimensional multimodal functions (F14–F23), both algorithms yield comparable mean performance on most functions. However, MIZOA consistently achieves lower standard deviations, reflecting superior solution stability. The multi-population search strategy adopted in MIZOA enhances the balance between exploration and exploitation, thereby improving the overall robustness of the algorithm.

In summary, MIZOA exhibits superior overall performance compared to MZOA, achieving a better trade-off between robustness and global convergence through the effective integration of multiple enhancement strategies.

**Figure 4:**
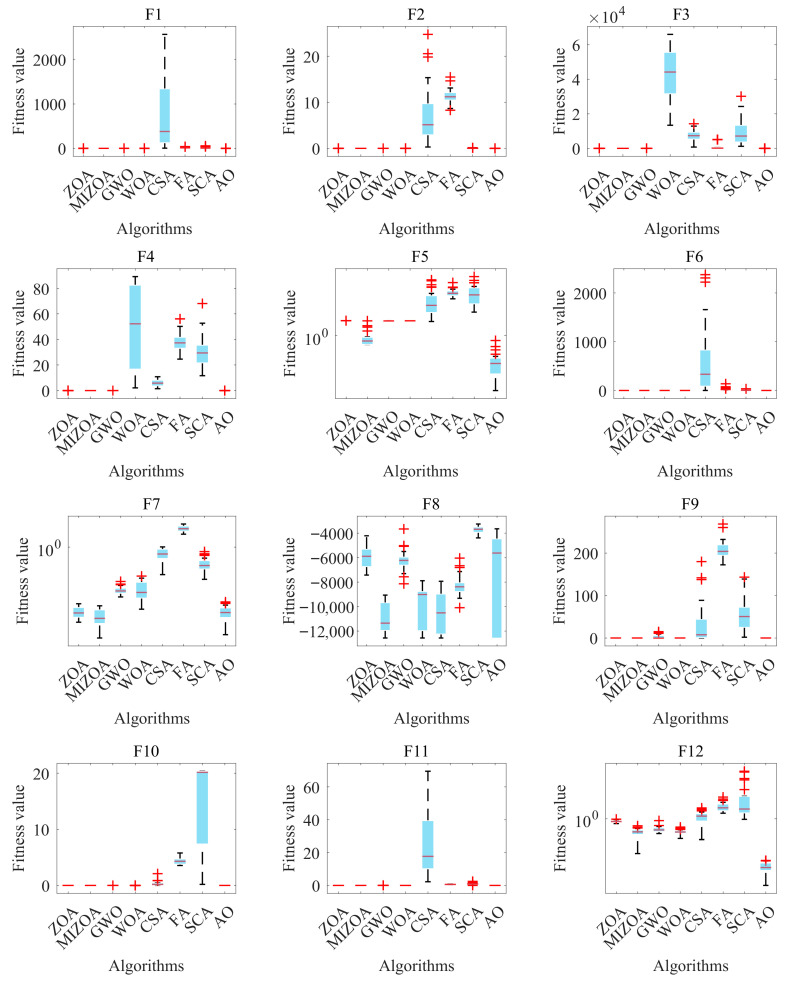

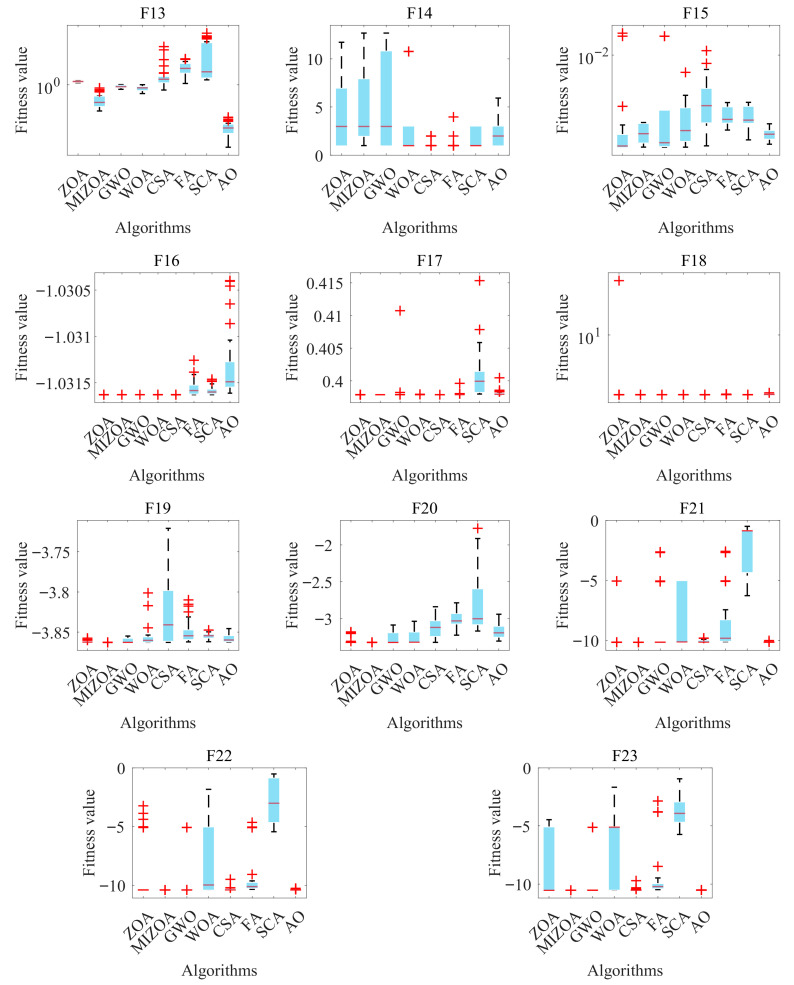
Box plot comparison of the algorithms on benchmark functions.

A box plot is a graphical tool commonly used to visualize the distribution of data, effectively revealing key characteristics such as central tendency, dispersion, and the presence of outliers across one or more datasets. The primary components of a box plot include the minimum value, first quartile (Q1), median (Q2), third quartile (Q3), maximum value, and any potential outliers. The interquartile range (IQR), represented by the height of the box, indicates the spread and concentration of the data. In this study, box plots are employed to illustrate the distribution of convergence results obtained from multiple independent runs on different benchmark test functions. This visualization provides an intuitive means to compare central tendencies, variability, and distribution patterns across the performances of various algorithms. Figure 4 presents the box plot distributions of the convergence results achieved by the eight optimization algorithms over the 23 benchmark functions. The figure enables a clear and direct comparison of each algorithm’s performance in terms of solution accuracy, consistency, and the presence of outliers, thereby offering valuable insights into their relative strengths and weaknesses.

As illustrated in Figure 4, the proposed MIZOA demonstrates notable performance advantages across the 23 benchmark functions. It consistently achieves lower median values and smaller interquartile ranges compared to the other algorithms, indicating superior performance in terms of both convergence accuracy and result stability. In addition, the occurrence rate of outliers in MIZOA is significantly lower than in the other algorithms, further highlighting the enhanced robustness and reliability contributed by the proposed improvement strategies. These experimental results provide strong and convincing evidence for the effectiveness and competitiveness of MIZOA in addressing complex, high-dimensional, and multimodal optimization problems.

### 4.5. Statistical Analysis

The Friedman test is a nonparametric statistical method employed to determine whether statistically significant differences exist among multiple related samples [56]. It is particularly well-suited for repeated-measures designs, where the same experimental subjects or units are evaluated under different conditions. In this study, the Friedman test is applied to assess whether the optimization results obtained for the same benchmark function by different algorithms differ significantly. As a nonparametric alternative to repeated-measures ANOVA, the Friedman test is especially appropriate when the data do not satisfy the parametric assumptions of ANOVA, such as normality and homogeneity of variances. This makes it a robust tool for comparative performance analysis in optimization studies, where result distributions often deviate from normality. The outcomes of the Friedman test for the evaluated algorithms are summarized in Table 8.

**Figure 5:**
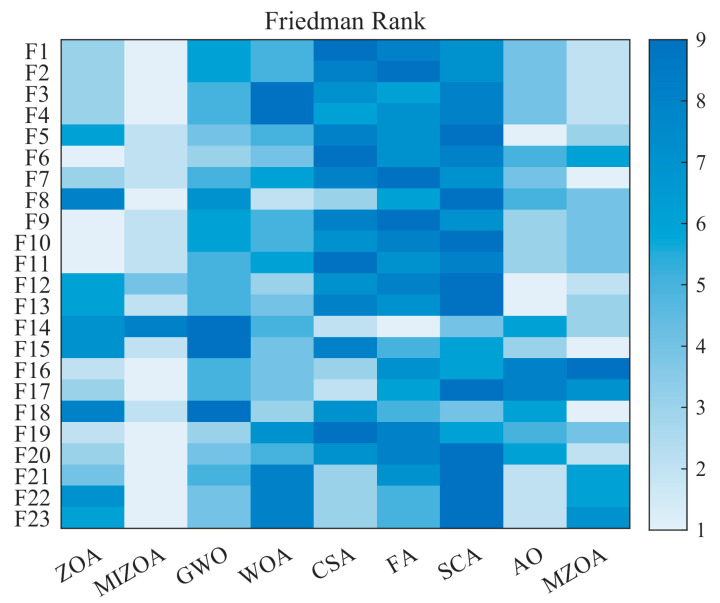
Heat map of the Friedman ranks of the algorithms on benchmark functions.

As shown in Table 8, MIZOA achieves the best Friedman ranking among the nine algorithms, with an average rank of 2.2, while MZOA ranks second with an average rank of 3.4. The corresponding *p*-value is far below 0.01, indicating that the optimization results obtained by MIZOA differ significantly from those of the other algorithms. These findings demonstrate that MIZOA outperforms both MZOA and the remaining seven algorithms in terms of overall performance, thereby further confirming the effectiveness and superiority of the proposed multi-strategy improvement framework. Additionally, a heatmap is utilized to visually represent the Friedman ranks of each algorithm across various test functions. As shown in Figure 5, for the majority of the functions, the cells corresponding to MIZOA exhibit lighter colors, indicating higher ranks. This visual representation confirms MIZOA’s superior overall performance across most functions.

The Wilcoxon rank sum test, also known as the Mann–Whitney U test, is a widely used nonparametric statistical method for comparing the distributions of two independent samples [57]. It is applied to determine whether the two samples are likely to originate from the same population distribution, thereby assessing whether a statistically significant difference exists between them. When the obtained *p*-value is less than the predefined significance level (set to 0.05 in this study), it indicates a significant difference between the two groups. Unlike parametric tests, the Wilcoxon rank sum test makes no assumptions about the underlying data distribution, making it particularly suitable for analyzing non-normally distributed datasets or those with small sample sizes. In this experiment, if the data distributions of two independent samples are identical, the corresponding *p*-value is set to 1. In Table 9, the symbol ‘+’ indicates that the *p*-value between the two algorithms is less than the significance threshold, implying a statistically significant difference in their performance. Conversely, the symbol ‘−’ denotes that the *p*-value exceeds the threshold, indicating no statistically significant difference between the two algorithms. The symbol ‘=’ signifies that the experimental results of the two algorithms are identical or highly similar.

For example, in the comparison between ZOA and MIZOA, the numbers of ‘+’, ‘−’, and ‘=’ were 18, 1, and 4, respectively. This indicates that, out of all test functions, there was one function where no statistically significant difference was observed between MIZOA and ZOA, and four functions where the results of both algorithms were identical or highly similar. As shown in Table 9, the *p*-values between the improved algorithm MIZOA and most of the compared algorithms are less than 0.05. This result indicates that the performance differences between MIZOA and these algorithms are statistically significant, further validating the effectiveness of the proposed improvement strategies and confirming the superior performance of MIZOA across the benchmark functions.

### 4.6. Comparison of Algorithm Runtime Performance

**Figure 6:**
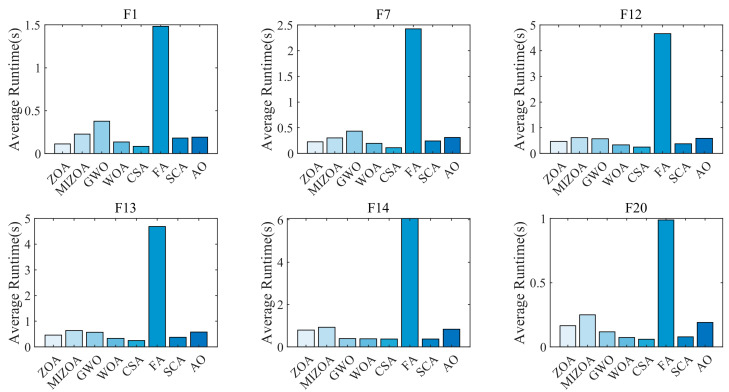
Runtime Comparison of Eight Algorithms on Six Benchmark Functions.

Given that real-time AGV systems require fast convergence and low computational latency, we quantitatively evaluate the runtime of each algorithm to assess its feasibility for real-time path planning. To comprehensively examine performance across different problem landscapes, six representative benchmark functions are selected: two unimodal, two multimodal, and two fixed-dimension multimodal functions. Parameter settings are consistent with those in Section 4.1. Each algorithm is independently run 30 times, and the average runtime is computed. The results are presented in Figure 6.

Figure 6 shows that FA consistently records the longest runtime across all test functions, exceeding those of other algorithms by several-fold. On F1, MIZOA’s runtime is approximately twice that of ZOA; however, this increase is accompanied by a substantial improvement in optimization accuracy, indicating a favorable trade-off between computational cost and solution quality. For the multimodal functions F12 and F13, MIZOA’s runtime is comparable to that of GWO and AO but remains slightly longer than that of the original ZOA, reflecting the computational overhead introduced by its enhanced search mechanisms. On the fixed-dimensional multimodal function F20, MIZOA’s average runtime is approximately 0.3 s—a 50% increase compared to ZOA (0.2 s)—due to the additional processing demands of its improved strategies. Overall, the marginally longer runtime of MIZOA, stemming from its multi-strategy enhancement framework, is well justified by its superior global convergence and solution quality, representing a balanced trade-off between computational efficiency and optimization performance.

### 4.7. Engineering Design Optimization Problems

To further evaluate the performance of the MIZOA on constrained optimization problems, this section considers three classical engineering design optimization problems: the tension/compression spring design, the pressure vessel design, and the speed reducer design. The performance of the MIZOA is compared against seven other metaheuristic algorithms under identical conditions. The results demonstrate the improved algorithm’s superior capability in handling complex constraints and achieving high-quality solutions, thereby validating the effectiveness of the proposed enhancements.

#### 4.7.1. Tension/Compression Spring Design Problem

**Figure 7:**
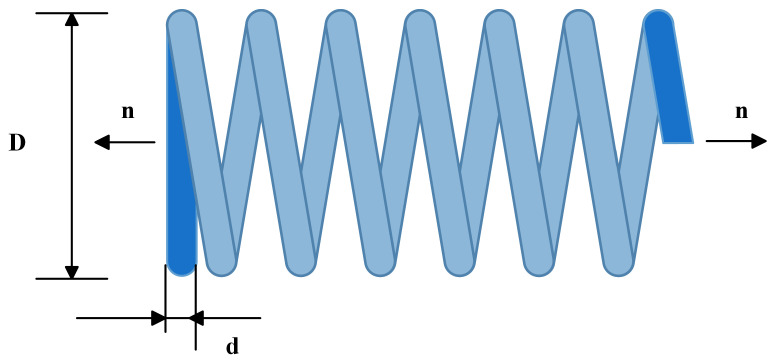
Schematic diagram of the tension/compression spring design variables.

The tension/compression spring design problem is a classical benchmark constrained optimization task in mechanical engineering. It involves three continuous decision variables: the mean coil diameter D, the wire diameter d, and the number of active coils n (Figure 7). The objective is to minimize the spring’s weight, subject to four constraints associated with deflection, shear stress, surge frequency, and geometric limitations. These constraints create a complex feasible region, making the problem a representative and challenging test case for metaheuristic algorithms. The mathematical formulation is presented in Equations (29)–(33).

(29) $$f(\vec{x})=(x_{3}+2)x_{2}x_{1}^{2}$$

(30) $$g_{1}(\vec{x})=1-\frac{x_{2}^{3}x_{3}}{71,785x_{1}^{4}}\leq 0$$

(31) $$g_{2}(\vec{x})=\frac{4x_{2}^{2}-x_{1}x_{2}}{12,566(x_{1}x_{3}^{3}-x_{1}^{4})}+\frac{1}{5108x_{1}^{2}}-1\leq 0$$

(32) $$g_{3}(\vec{x})=1-\frac{140.45x_{1}}{x_{2}^{2}x_{3}}\leq 0$$

(33) $$g_{4}(\vec{x})=\frac{x_{1}+x_{2}}{1.5}-1\leq 0$$

Here, *f*(*x*) denotes the objective function to be minimized. $\vec{x}=[x_{1},x_{2},x_{3}]$,$x_{1}\in [0.05,2],x_{2}\in [0.25,1.3],x_{3}\in [2,15]$. 

Table 10 shows that MIZOA achieves the lowest average fitness value, representing a 6% improvement over the traditional ZOA in terms of mean solution quality. The standard deviation of MIZOA is 6.333 × 10^−05^, indicating significantly enhanced stability. These results further validate the effectiveness of the improved algorithm and demonstrate its superior performance in solving constrained spring design optimization problems.

#### 4.7.2. Speed Reducer Design Problem

**Figure 8:**
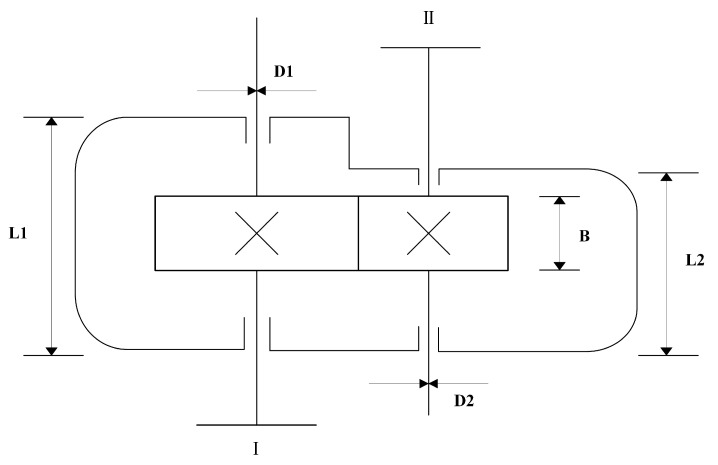
Schematic diagram of the speed reducer showing design variables.

The speed reducer is a critical component in various mechanical systems and is widely used in industrial machinery. A schematic of the system is illustrated in Figure 8. Compared to the spring design problem, this optimization problem is more complex, involving seven design variables and eleven nonlinear constraints (see Equations (35)–(45)). The design variables are defined as follows: *x*_1_: gear face width *B*, *x*_2_: gear module *m*, *x*_3_: number of pinion teeth *z*, *x*_4_: distance between bearings on the input shaft *L1*, *x*_5_: distance between bearings on the output shaft *L2*, *x*_6_: diameter of the input shaft *D1*, *x*_7_: diameter of the output shaft *D2*. The objective is to minimize the total weight of the reducer, expressed by the objective function in Equation (34).

(34) $$f(\vec{x})=0.7854x_{1}x_{2}^{2}(3.3333x_{3}^{2}+14.9334x_{3}-43.0934)-1.508x_{1}(x_{6}^{2}+x_{7}^{2})+7.4777(x_{6}^{3}+x_{7}^{3})+0.7854(x_{4}x_{6}^{2}+x_{5}x_{7}^{2})$$

(35) $$g_{1}(\vec{x})=\frac{27}{x_{1}x_{2}^{2}x_{3}}-1\leq 0$$

(36) $$g_{2}(\vec{x})=\frac{397.5}{x_{1}x_{2}^{2}x_{3}^{2}}-1\leq 0$$

(37) $$g_{3}(\vec{x})=\frac{1.93x_{4}^{3}}{x_{2}x_{6}^{4}x_{3}}-1\leq 0$$

(38) $$g_{4}(\vec{x})=\frac{1.93x_{5}^{3}}{x_{2}x_{7}^{4}x_{3}}-1\leq 0$$

(39) $$g_{5}(\vec{x})=\frac{{\left[{\left(745(x_{4}/x_{2}x_{3})\right)}^{2}+16.9\times 10^{6}\right]}^{1/2}}{110x_{6}^{3}}-1\leq 0$$

(40) $$g_{6}(\vec{x})=\frac{{\left[{\left(745(x_{5}/x_{2}x_{3})\right)}^{2}+157.5\times 10^{6}\right]}^{1/2}}{85x_{7}^{3}}-1\leq 0$$

(41) $$g_{7}(\vec{x})=\frac{x_{2}x_{3}}{40}-1\leq 0$$

(42) $$g_{8}(\vec{x})=\frac{5x_{2}}{x_{1}}-1\leq 0$$

(43) $$g_{9}(\vec{x})=\frac{x_{1}}{12x_{2}}-1\leq 0$$

(44) $$g_{10}(\vec{x})=\frac{1.5x_{6}+1.9}{x_{4}}-1\leq 0$$

(45) $$g_{11}(\vec{x})=\frac{1.1x_{7}+1.9}{x_{5}}-1\leq 0$$

where $\vec{x}=[x_{1},x_{2},x_{3},x_{4},x_{5},x_{6},x_{7}]$, $2.6\leq x_{1}\leq 3.6$, $0.7\leq x_{2}\leq 0.8$, $17\leq x_{3}\leq 28$, $7.3\leq x_{4}\leq 8.3$, $7.8\leq x_{5}\leq 8.3$, $2.9\leq x_{6}\leq 3.9$, $5.0\leq x_{7}\leq 5.5$.

Table 11 shows that the improved algorithm achieves the best performance across all four metrics: mean, standard deviation, optimal value, and worst value. MIZOA obtains a mean fitness of 2.995 × 10^+03^ and a standard deviation of 0.83. Compared to the original ZOA, the standard deviation is reduced by approximately two orders of magnitude, indicating significantly enhanced solution stability. The difference between MIZOA’s best and worst results is only 4 units, further demonstrating its robustness. These results validate the improved algorithm’s superior convergence performance in solving constrained engineering optimization problems. Table 12 presents the optimal design variable values obtained by each algorithm.

#### 4.7.3. Pressure Vessel Design Problem

**Figure 9:**
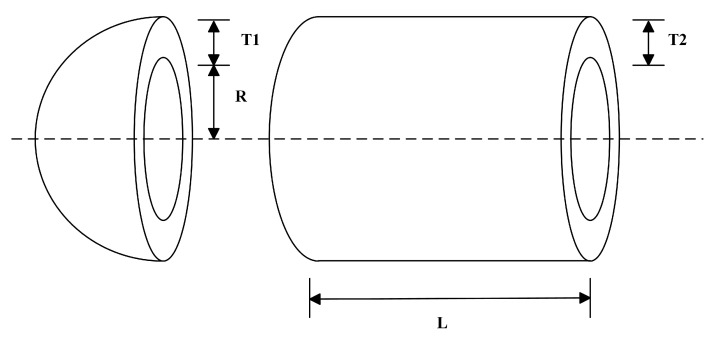
Schematic diagram of the pressure vessel showing design variables.

The pressure vessel design problem is a benchmark in constrained engineering optimization, frequently used to evaluate the performance of metaheuristic algorithms. The objective is to minimize manufacturing cost while satisfying strength, geometric, and technological constraints. As shown in Figure 9, the vessel consists of a cylindrical shell capped with hemispherical heads. The problem involves four design variables: *x*_1_: thickness of the cylindrical shell *T2*, *x*_2_: thickness of the hemispherical head *T1*, *x*_3_: inner radius of the vessel *R*, *x*_4_: length of the cylindrical section *L*. The objective function is defined in Equation (46), and the constraints are given in Equations (47)–(50).

(46) $$f(\vec{x})=0.6224x_{1}x_{3}x_{4}+1.778x_{2}x_{3}^{2}+3.1661x_{1}^{2}x_{4}+19.84x_{1}^{2}x_{3}$$

(47) $$g_{1}(\vec{x})=-x_{1}+0.0193x_{3}\leq 0$$

(48) $$g_{2}(\vec{x})=-x_{2}+0.00954x_{3}\leq 0$$

(49) $$g_{3}(\vec{x})=-\pi x_{3}^{2}x_{4}-\frac{4}{3}\pi x_{3}^{3}+1,296,000\leq 0$$

(50) $$g_{4}(\vec{x})=x_{4}-240\leq 0$$

(51) $$0\leq x_{1},x_{2}\leq 99$$

(52) $$10\leq x_{3},x_{4}\leq 200$$

Table 13 presents the statistical results of eight algorithms on the pressure vessel design problem. The MIZOA achieves the best mean value of 1.406 × 10^2^, outperforming the original ZOA by approximately one order of magnitude. It also exhibits a significantly lower standard deviation, indicating improved solution consistency. Furthermore, the MIZOA obtains the best and the closest worst solution among all compared algorithms, demonstrating its superior robustness. These results confirm that the proposed improvements effectively enhance the algorithm’s performance in constrained optimization. The optimal design variable values are presented in Table 14.

The proposed algorithm exhibits consistent and robust performance across multiple benchmarks engineering problems, including the pressure vessel, speed reducer, and tension/compression spring design problems. This consistency highlights the effectiveness of the integrated enhancement strategies—Multi-Population Search, Mutation Operation, Metropolis Criterion, and Selective Aggregation—in maintaining a balance between exploration and exploitation and in preserving feasibility under nonlinear constraints.

## 5. AGV Path Planning

An AGV is an intelligent mobile platform capable of autonomously performing material handling tasks without human intervention. It is widely employed in modern manufacturing systems, logistics warehousing, and smart factory environments, among other automated operational scenarios. AGV offer significant advantages such as autonomous navigation, high flexibility, and robust operational safety, enabling them to carry out material transportation and distribution tasks along predefined paths in complex environments without manual guidance. With the continuous development of Industry 4.0 and smart manufacturing paradigms, AGV have become essential components in intelligent logistics and flexible manufacturing systems. Within AGV systems, path planning serves as one of the core functionalities, and its effectiveness directly influences system efficiency, safety, and overall operational stability [58].

Numerous path planning methods have been developed for AGV, including graph search-based approaches, sampling-based techniques, geometric curve-based methods, and metaheuristic algorithm-based strategies. Among these, metaheuristic algorithm-based approaches exhibit notable advantages in handling complex, dynamic, and large-scale environments due to their superior global search capabilities and adaptability [59]. In real-world workshop environments, AGV are required to generate feasible paths between specified start and end points, with the primary objective being the shortest possible route while ensuring effective obstacle avoidance [60,61]. To evaluate the performance of MIZOA in AGV path planning, comparative experiments were conducted against several established optimization algorithms, including ZOA, AO, WOA, CSA, and SCA.

### 5.1. Experimental Setup for Path Planning

**Figure 10:**
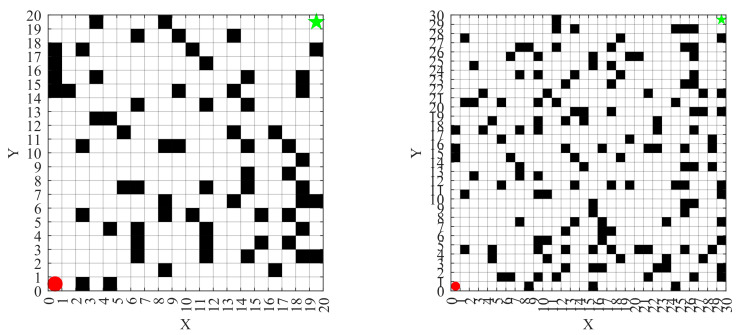
Two Grid Maps for Path Planning with Static Obstacles.

In this study, a grid-based map is employed to model the real-world workshop environment for AGV navigation. As illustrated in Figure 10, black cells represent obstacles, while white cells indicate free spaces through which the AGV can move. The starting point is located at the bottom-left corner of the map, and the target point is positioned at the top-right corner, simulating a typical point-to-point navigation task in industrial production environments.

To evaluate path planning performance under different levels of environmental complexity, two types of grid maps are designed:

- A simple environment consisting of a 20 × 20 grid with a sparse distribution of obstacles.
- A complex environment based on a 30 × 30 grid, featuring a higher obstacle density and a more intricate spatial arrangement, providing a realistic representation of the constraints and challenges commonly encountered in industrial workshop layouts.

During the path search process, the AGV is allowed to move in eight possible directions: up, down, left, right, and the four diagonals. This multi-directional movement strategy enhances the AGV’s flexibility in navigating complex environments and contributes to generating smoother, more efficient paths.

### 5.2. Fitness Function

In this experiment, the paths of the AGV are encoded within each individual of the optimization population. Specifically, each individual represents a feasible path, with the dimensionality of the individual corresponding to the number of path points. This encoding scheme enables the algorithm to effectively explore a wide range of path configurations throughout the optimization process. The mathematical formulation of the path encoding is presented in Equations (53)–(55)

(53) $$X_{i}=\{xl_{i,1},xl_{i,2},xl_{i,3},\ldots ,xl_{i,m}\}+X_{i,sub}$$

(54) $$x_{i}=\{round(linspace(1:n:m))\}+x_{i,sub}$$

(55) $$y_{i}=y_{initial}+y_{i,sub}$$

Among them, *X_i_* denotes the *i*-th path, and *X_i,sub_* represents the interpolated path within it. Specifically, *x* represents the sequence of horizontal coordinates along the path, while *y* represents the corresponding sequence of vertical coordinates. The function *linspace* (*1*: *n*: *m*) generates an arithmetic sequence from 1 to *n* containing *m* equally spaced elements, where *n* is the total number of cells in the horizontal direction of the grid map. The *round* function is applied to convert continuous values into integer values, ensuring that the generated path points correspond to valid grid cell indices.

In the experiment, the fitness function is defined as the total length of the planned path, which is computed as the sum of the Euclidean distances between all consecutive nodes along the path. This ensures that shorter and more efficient paths yield lower fitness values. The mathematical formulation for calculating the total path length is provided in Equation (56).

(56) $$L_{i}=\sum_{j=1}^{m-1}\sqrt{{\left(x_{j}-x_{j+1}\right)}^{2}+{\left(y_{j}-y_{j+1}\right)}^{2}}$$

where *L_i_* represents the total length of the path for the *i*th individual, m denotes the number of path points (i.e., the dimension of the individual), *x_j_* is the horizontal coordinate of the *j*th path point, and *y_j_* is the corresponding vertical coordinate.

### 5.3. Simulation Experiment

In this experiment, the population size was set to 50, and the maximum number of iterations was limited to 500. Each algorithm was independently executed 30 times, and the mean, standard deviation, and best-obtained solution were recorded for performance evaluation. The final experimental results are summarized in Table 15.

As shown in Table 15, on a 20 × 20 grid map, MIZOA achieved the best performance in both the average and best path lengths. Specifically, MIZOA attained a mean path length of 27.84, representing an improvement of approximately 19% over the original ZOA. Although its stability is lower than that of CSA, MIZOA shows a significant improvement compared to ZOA and ranks second among all compared algorithms in terms of solution consistency (i.e., lowest standard deviation). For a more comprehensive evaluation, we also include the recently proposed MZOA. While MZOA achieves a best path length comparable to MIZOA, its mean path length is approximately 3 units higher over 30 independent runs. Furthermore, MZOA exhibits a larger standard deviation (e.g., σ = 0.39 vs. MIZOA’s σ = 1.12), indicating lower stability and less consistent performance across runs.

On a more complex 30 × 30 grid map, MIZOA demonstrates superior performance compared to all competing algorithms, achieving the best results in both the minimum (best) and mean path lengths. While MZOA attains a comparable optimal path length, MIZOA yields a significantly lower mean path length and a smaller standard deviation, indicating enhanced solution consistency and robustness across multiple runs. Specifically, MIZOA improves the average path length by approximately 15% over the original ZOA, highlighting its strong adaptability and excellent optimization capability in both simple and complex environments. This enhanced performance is attributed to the integration of multi-population search strategies, mutation operations, and the Metropolis acceptance criterion, which collectively enable MIZOA to effectively escape local optima and achieve faster convergence toward high-quality solutions. Overall, MIZOA outperforms MZOA in terms of convergence accuracy, solution quality, and algorithmic stability, thereby validating the effectiveness of the proposed improvements.

**Figure 11:**
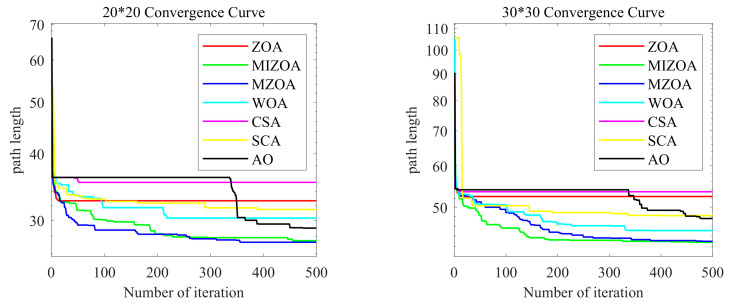
Convergence Curves of Different Algorithms for Path Planning.

Figure 11 compare the convergence curves of the seven algorithms on 20 × 20 and 30 × 30 grid maps, respectively. MIZOA demonstrates strong convergence performance across both environments, achieving the fastest convergence speed and among the best final solutions, ranking first in overall performance. On the 20 × 20 map, MZOA exhibits faster initial convergence in the first 100 iterations. However, MIZOA gradually closes the gap, and by the 200th iteration, both algorithms show similar convergence rates. In the later stages, their convergence speeds are comparable, and they achieve nearly identical final objective values, indicating similar exploitation capabilities. On the more complex 30 × 30 map, MIZOA consistently outperforms MZOA in convergence speed throughout the optimization process. During the first 500 iterations, MIZOA maintains a rapid and stable descent in the objective value, surpassing all other algorithms, including MZOA. The results indicate that the proposed selective aggregation strategy achieves an effective balance between global exploration and local exploitation. By maintaining population diversity in the early stages to avoid premature convergence, and by accelerating convergence in the later stages, the strategy ensures a dynamic balance that enhances MIZOA’s adaptability and robustness across diverse environmental complexities.

**Figure 12:**
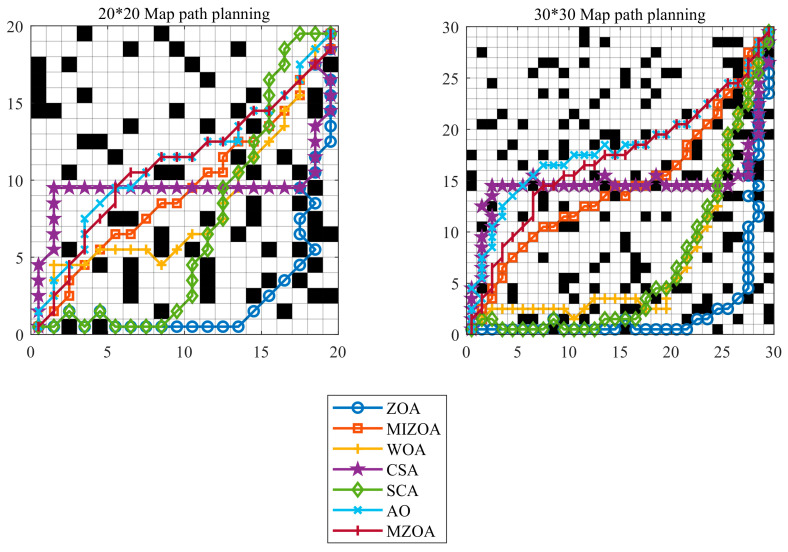
Comparison of Path Planning Results for Different Algorithms.

Figure 12 illustrates the path planning results of the seven algorithms on 20 × 20 and 30 × 30 grid maps. Visually, the path generated by MIZOA is the closest to the ideal straight-line (diagonal) trajectory, indicating high solution quality with minimal detours. On the 20 × 20 map, MZOA produces a slightly longer path due to a mid-course deviation, resulting in an unnecessary detour. A similar behavior is observed in the 30 × 30 environment, where MZOA again deviates from the optimal direction, leading to a markedly increased path length. In both scenarios, MIZOA consistently generates shorter and more direct paths, demonstrating its enhanced global search capability and robustness across varying environmental complexities. Such improved performance enables automated guided vehicles (AGV) to navigate more efficiently in complex and dynamic industrial environments, providing reliable support for real-world path planning applications.

**Figure 13:**
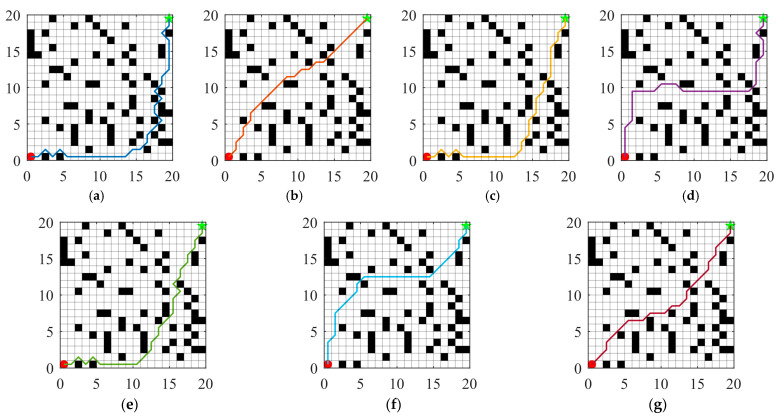
Trajectory Visualization of Path Planning by a Single Algorithm in a 20 × 20 Environment: (**a**) ZOA; (**b**) MIZOA; (**c**) WOA; (**d**) CSA; (**e**) SCA: (**f**) AO; (**g**) MZOA.

**Figure 14:**
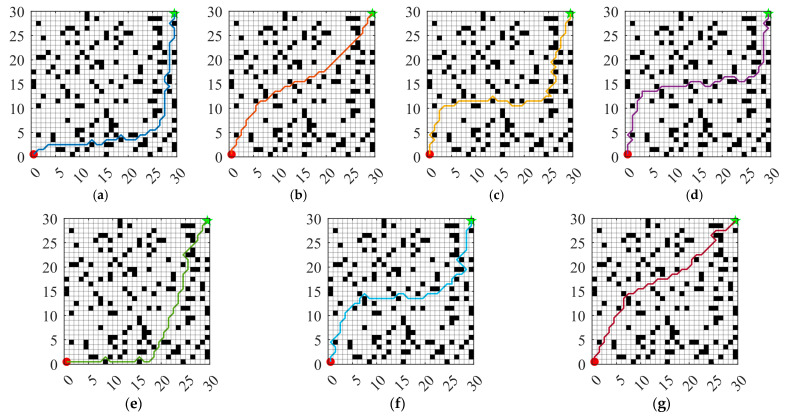
Trajectory Visualization of Path Planning by a Single Algorithm in a 30 × 30 Environment: (**a**) ZOA; (**b**) MIZOA; (**c**) WOA; (**d**) CSA; (**e**) SCA: (**f**) AO; (**g**) MZOA.

Figure 13 and Figure 14 illustrate the planned trajectories of the seven algorithms on 20 × 20 and 30 × 30 grid maps, respectively. On the simple 20 × 20 map, ZOA, AO, and CSA generate highly tortuous paths with frequent directional changes, indicating limited exploration and a tendency toward premature convergence. SCA and WOA produce somewhat more direct routes but still exhibit noticeable detours. MZOA maintains a generally correct direction but suffers from mid-path deviations and local oscillations, resulting in a less efficient trajectory. In contrast, MIZOA generates the smoothest and most direct path, with minimal turns and the least deviation from the ideal diagonal line, reflecting superior path quality and convergence behavior. On the more complex 30 × 30 map, a similar trend is observed. Most algorithms, including MZOA, exhibit increased path tortuosity and directional instability. While MZOA follows the general direction toward the goal, its path contains numerous small bends, reducing smoothness and efficiency. Only MIZOA consistently maintains a straight and stable trajectory, closely aligned with the diagonal reference, indicating robust performance under environmental complexity. These results demonstrate that the multi-strategy enhancements in MIZOA—particularly multi-population search strategies, mutation operations, and the Metropolis acceptance criterion—effectively balance global exploration and local exploitation. This enables the algorithm to avoid local traps, maintain directional consistency, and converge toward high-quality, near-optimal paths. The improved trajectory quality further demonstrates MIZOA’s strong potential for practical AGV path planning in complex industrial environments.

## 6. Conclusions and Future Work

To address the limitation of the traditional ZOA in easily becoming trapped in local optima, this paper proposes a multi-strategy improved variant, termed the MIZOA. The proposed algorithm demonstrates substantial performance improvements in simulation experiments. The MIZOA integrates several complementary enhancement strategies, including multi-population search, genetic algorithm-based mutation operation, the Metropolis acceptance criterion, and a selective aggregation strategy. Specifically, the multi-population search mechanism partitions the original single population into multiple subpopulations, effectively reducing the risk of premature convergence caused by dominant pioneer zebras and preserving population diversity. The incorporation of a genetic mutation operation introduces additional randomness into the search process, further enhancing diversity and increasing the likelihood of achieving global convergence. The adoption of the Metropolis criterion as the acceptance rule for new positions during mutation allows suboptimal solutions to be probabilistically accepted, thereby improving the algorithm’s global exploration capability and facilitating escape from local optima. Furthermore, the selective aggregation strategy integrates Lévy flight with the COA, enhancing the algorithm’s global exploration ability during the defense phase while maintaining effective local exploitation. In the experimental study, the performance of eight optimization algorithms was comprehensively evaluated on a diverse set of benchmark functions. The results were visualized through convergence curves and box plots and further validated via statistical analyses, including the Wilcoxon rank-sum test and the Friedman test. Both experimental and statistical results demonstrate that the MIZOA consistently outperforms the other comparative algorithms in terms of overall performance. Additionally, when applied to AGV path planning problems, the MIZOA successfully generated paths closest to the global optimal solution, achieving the highest rank among all compared algorithms and demonstrating superior path planning performance.

Nevertheless, while the MIZOA exhibits strong overall performance across most benchmark functions and path planning environments, it may not guarantee optimal outcomes for all types of optimization problems. Therefore, there remains significant potential for further enhancement to improve its adaptability and generalization capability. Future research could explore the integration of additional advanced algorithmic components or hybrid frameworks to further strengthen its performance. Additionally, as the MIZOA incurs slightly higher computational complexity compared to the original ZOA, subsequent studies could focus on optimizing computational efficiency and reducing algorithmic overhead to enhance scalability in large-scale and real-time optimization applications. In certain optimization domains, modern deterministic mathematical optimization methods also possess distinct advantages. In future work, we plan to conduct further comparative analyses involving these methods.

## Figures and Tables

**Table 1:** Unimodal benchmark functions.

**Function**	**Dim (*m*)**	**Range (*x_i_*)**	**Optimal**
$F_{1}(X)=\sum_{i=1}^{m}x_{i}^{2}$	30	[−100, 100]	0
$F_{2}(X)=\sum_{i=1}^{m}\left|x_{i}\right|+\prod_{i=1}^{m}\left|x_{i}\right|$	30	[−10, 10]	0
$F_{3}(X)=\sum_{i=1}^{m}{\left(\sum_{j=1}^{i}x_{j}\right)}^{2}$	30	[−100, 100]	0
$F_{4}(X)=\max_{i}\{|x_{i}|,1\leq i\leq m\}$	30	[−100, 100]	0
$F_{5}(x)=\sum_{i=1}^{m-1}\left[100{\left(x_{i+1}-x_{i}^{2}\right)}^{2}+{\left(x_{i}-1\right)}^{2}\right]$	30	[−30, 30]	0
$F_{6}(x)=\sum_{i=1}^{m}(\left\lfloor x_{i}+0.5\right\rfloor {)}^{2}$	30	[−100, 100]	0
$F_{7}(x)=\sum_{i=1}^{m}ix_{i}^{4}+\mathrm{random}[0,1)$	30	[−1.28, 1.28]	0

**Table 2:** Multimodal benchmark functions.

Function	Dim (*m*)	Range (*x_i_*)	Optimal
$F_{8}(X)=\sum_{i=1}^{m}-x_{i}\sin(\sqrt{\left|x_{i}\right|})$	30	[−500, 500]	428.989×m
$F_{9}(X)=\sum_{i=1}^{m}\left[x_{i}^{2}-10\cos(2\pi x_{i})+10\right]$	30	[−5.12, 5.12]	0
$F_{10}(X)=-20\exp\left(-0.2\sqrt{\frac{1}{m}\sum_{i=1}^{m}x_{i}^{2}}\right)-\exp\left(\frac{1}{m}\sum_{i=1}^{m}\cos(2\pi x_{i})\right)+20+e$	30	[−32, 32]	0
$F_{11}(X)=\frac{1}{4000}\sum_{i=1}^{m}x_{i}^{2}-\prod_{i=1}^{m}\cos\left(\frac{x_{i}}{\sqrt{i}}\right)+1$	30	[−600, 600]	0
$\begin{array}{l} F_{12}(x)=\frac{\pi }{m}\left\{10\sin^{2}(\pi y_{i})+\sum_{i=1}^{n-1}{\left(y_{i}-1\right)}^{2}[1+10\sin^{2}(\pi y_{i+1})]+{\left(y_{n}-1\right)}^{2}\right\}\\ +\sum_{i=1}^{m}u(x_{i},10,100,4) \end{array}$ $y_{i}=1+\frac{1}{4}(x_{i}+1)$, $u(x_{i},a,k,l)=\left\{\begin{array}{ll} k{\left(x_{i}-a\right)}^{l}, & x_{i}>a,\\ 0, & -a\leq x_{i}\leq a,\\ k{\left(-x_{i}-a\right)}^{l}, & x_{i}<-a. \end{array}\right.$	30	[−50, 50]	0
$\begin{array}{l} F_{13}(x)=0.1\left\{\sin^{2}(3\pi x_{1})+\sum_{i=1}^{m-1}{\left(x_{i}-1\right)}^{2}[1+\sin^{2}(3\pi x_{i+1})]+{\left(x_{m}-1\right)}^{2}[1+\sin^{2}(2\pi x_{m})]\right\}\\ +\sum_{i=1}^{m}u(x_{i},5,100,4) \end{array}$	30	[−50, 50]	0

**Table 3:** Fixed dimension multimodal benchmark functions.

Function	Dim (*m*)	Range (*x_i_*)	Optimal
$F_{14}(x)={\left[\frac{1}{500}+\sum_{j=1}^{25}\frac{1}{j+\sum_{i=1}^{2}{\left(x_{i}-a_{ij}\right)}^{6}}\right]}^{-1}$ $a_{ij}=\left(\begin{array}{cccccccccc} -32 & -16 & 0 & 16 & 32 & -32 & \ldots & 0 & 16 & 32\\ -32 & -32 & -32 & -32 & -32 & -16 & \ldots & 32 & 32 & 32 \end{array}\right).$	2	[−65.536, 65.536]	1
$F_{15}(x)=\sum_{i=1}^{11}{\left[a_{i}-\frac{x_{1}(b_{i}^{2}+b_{i}x_{2})}{b_{i}^{2}+b_{i}x_{3}+x_{4}}\right]}^{2}$	4	[−5, 5]	0.0003075
$F_{16}=4x_{1}^{2}-2.1x_{4}^{4}+\frac{1}{3}x_{1}^{6}+x_{1}x_{2}-4x_{2}^{2}+4x_{2}^{4},$	2	[−5, 5]	−1.0316285
$F_{17}(x)={\left(x_{2}-\frac{5.1}{4\pi^{2}}x_{1}^{2}+\frac{5}{\pi }x_{1}-6\right)}^{2}+10\left(1-\frac{1}{8\pi }\right)\cos x_{1}+10$	2	[−5, 10], [0, 15]	0.398
$\begin{array}{l} F_{18}(x)=\left[1+{\left(x_{1}+x_{2}+1\right)}^{2}\left(19-14x_{1}+3x_{1}^{2}-14x_{2}+6x_{1}x_{2}+3x_{2}^{2}\right)\right]\\ \times \left[30+{\left(2x_{1}-3x_{2}\right)}^{2}(18-32x_{1}+12x_{1}^{2}+48x_{2}-36x_{1}x_{2}+27x_{2}^{2})\right] \end{array}$	2	[−2, 2]	3
$F_{19}(X)=-\sum_{i=1}^{4}c_{i}\exp\left(-\sum_{j=1}^{3}a_{ij}{\left(x_{j}-p_{ij}\right)}^{2}\right)$	3	[0, 1]	−3.86
$F_{20}(X)=-\sum_{i=1}^{4}c_{i}\exp\left(-\sum_{j=1}^{6}a_{ij}{\left(x_{j}-p_{ij}\right)}^{2}\right)$	6	[0, 1]	−3.32
$F_{21}(X)=-\sum_{i=1}^{5}{\left[(x-a_{i}){\left(x-a_{i}\right)}^{T}+c_{i}\right]}^{-1}$	4	[0, 10]	−10.1532
$F_{22}(X)=-\sum_{i=1}^{7}{\left[(x-a_{i}){\left(x-a_{i}\right)}^{T}+c_{i}\right]}^{-1}$	4	[0, 10]	−10.4029
$F_{23}(X)=-\sum_{i=1}^{10}{\left[(x-a_{i}){\left(x-a_{i}\right)}^{T}+c_{i}\right]}^{-1}$	4	[0, 10]	−10.5364

**Table 4:** Parameter settings for algorithms.

Algorithm	Parameters	Value
ZOA	*R*	0.01
	*I*	{1, 2}
MIZOA	*R*	0.01
	*k*	5
GWO	*a*	Linear decrease from 2 to 0
WOA	*b*	1
	*l*	[−1, 1]
	*a*	Linear decrease from 2 to 0
CSA	ρ	1.0
	*c*1, *c*2	2.0, 1.8
FA	$\beta_{0}$	2.0
	γ	1.0
	α	0.2
SCA	*a*	2
	$r_{2}$	2π×rand
	$r_{3}$	2×rand
AO	α,δ	0.1, 0.1
	$r_{0}$	10

**Table 5:** Different parameter combinations.

Scenario No.	Mutation Probability	k (Number of Subpopulations)
1	0.05	3
2	0.05	5
3	0.05	7
4	0.1	3
5	0.1	5
6	0.1	7
7	0.2	3
8	0.2	5
9	0.2	7

**Table 6:** Performance of Various Parameter Combinations on Benchmark Functions.

Fun No. Metric	Scenario No.								
	Scenario 1	Scenario 2	Scenario 3	Scenario 4	Scenario 5	Scenario 6	Scenario 7	Scenario 8	Scenario 9
F1									
AVG	0.00 × 10^+00^	0.00 × 10^+00^	0.00 × 10^+00^	0.00 × 10^+00^	0.00 × 10^+00^	0.00 × 10^+00^	0.00 × 10^+00^	0.00 × 10^+00^	0.00 × 10^+00^
STD	0.00 × 10^+00^	0.00 × 10^+00^	0.00 × 10^+00^	0.00 × 10^+00^	0.00 × 10^+00^	0.00 × 10^+00^	0.00 × 10^+00^	0.00 × 10^+00^	0.00 × 10^+00^
F2									
AVG	0.00 × 10^+00^	0.00 × 10^+00^	0.00 × 10^+00^	0.00 × 10^+00^	0.00 × 10^+00^	0.00 × 10^+00^	0.00 × 10^+00^	0.00 × 10^+00^	0.00 × 10^+00^
STD	0.00 × 10^+00^	0.00 × 10^+00^	0.00 × 10^+00^	0.00 × 10^+00^	0.00 × 10^+00^	0.00 × 10^+00^	0.00 × 10^+00^	0.00 × 10^+00^	0.00 × 10^+00^
F3									
AVG	0.00 × 10^+00^	0.00 × 10^+00^	0.00 × 10^+00^	0.00 × 10^+00^	0.00 × 10^+00^	0.00 × 10^+00^	0.00 × 10^+00^	0.00 × 10^+00^	0.00 × 10^+00^
STD	0.00 × 10^+00^	0.00 × 10^+00^	0.00 × 10^+00^	0.00 × 10^+00^	0.00 × 10^+00^	0.00 × 10^+00^	0.00 × 10^+00^	0.00 × 10^+00^	0.00 × 10^+00^
F4									
AVG	0.00 × 10^+00^	0.00 × 10^+00^	0.00 × 10^+00^	0.00 × 10^+00^	0.00 × 10^+00^	0.00 × 10^+00^	8.04 × 10^−51^	0.00 × 10^+00^	0.00 × 10^+00^
STD	0.00 × 10^+00^	0.00 × 10^+00^	0.00 × 10^+00^	0.00 × 10^+00^	0.00 × 10^+00^	0.00 × 10^+00^	4.40 × 10^−50^	0.00 × 10^+00^	0.00 × 10^+00^
F5									
AVG	1.83 × 10^+01^	2.55 × 10^+00^	6.91 × 10^−01^	1.61 × 10^+01^	1.03 × 10^+00^	2.55 × 10^−01^	1.18 × 10^+01^	2.17 × 10^+00^	4.18 × 10^−01^
STD	1.20 × 10^+01^	3.98 × 10^+00^	1.44 × 10^+00^	1.25 × 10^+01^	5.21 × 10^+00^	1.86 × 10^−01^	1.25 × 10^+01^	5.91 × 10^+00^	8.15 × 10^−01^
F6									
AVG	0.00 × 10^+00^	0.00 × 10^+00^	0.00 × 10^+00^	0.00 × 10^+00^	0.00 × 10^+00^	0.00 × 10^+00^	0.00 × 10^+00^	0.00 × 10^+00^	0.00 × 10^+00^
STD	0.00 × 10^+00^	0.00 × 10^+00^	0.00 × 10^+00^	0.00 × 10^+00^	0.00 × 10^+00^	0.00 × 10^+00^	0.00 × 10^+00^	0.00 × 10^+00^	0.00 × 10^+00^
F7									
AVG	5.19 × 10^−05^	7.41 × 10^−05^	4.33 × 10^−05^	5.28 × 10^−05^	6.91 × 10^−05^	6.55 × 10^−05^	8.58 × 10^−05^	8.32 × 10^−05^	7.17 × 10^−05^
STD	4.94 × 10^−05^	7.70 × 10^−05^	3.30 × 10^−05^	4.54 × 10^−05^	6.20 × 10^−05^	4.70 × 10^−05^	7.21 × 10^−05^	7.71 × 10^−05^	7.35 × 10^−05^
F8									
AVG	−1.04 × 10^+04^	−1.07 × 10^+04^	−1.05 × 10^+04^	−1.04 × 10^+04^	−1.10 × 10^+04^	−1.13 × 10^+04^	−1.08 × 10^+04^	−1.14 × 10^+04^	−1.17 × 10^+04^
STD	1.48 × 10^+03^	1.38 × 10^+03^	1.61 × 10^+03^	1.44 × 10^+03^	1.27 × 10^+03^	1.21 × 10^+03^	1.39 × 10^+03^	1.16 × 10^+03^	1.09 × 10^+03^
F9									
AVG	0.00 × 10^+00^	0.00 × 10^+00^	0.00 × 10^+00^	0.00 × 10^+00^	0.00 × 10^+00^	0.00 × 10^+00^	0.00 × 10^+00^	0.00 × 10^+00^	0.00 × 10^+00^
STD	0.00 × 10^+00^	0.00 × 10^+00^	0.00 × 10^+00^	0.00 × 10^+00^	0.00 × 10^+00^	0.00 × 10^+00^	0.00 × 10^+00^	0.00 × 10^+00^	0.00 × 10^+00^
F10									
AVG	4.44 × 10^−16^	4.44 × 10^−16^	4.44 × 10^−16^	4.44 × 10^−16^	4.44 × 10^−16^	4.44 × 10^−16^	4.44 × 10^−16^	4.44 × 10^−16^	4.44 × 10^−16^
STD	0.00 × 10^+00^	0.00 × 10^+00^	0.00 × 10^+00^	0.00 × 10^+00^	0.00 × 10^+00^	0.00 × 10^+00^	0.00 × 10^+00^	0.00 × 10^+00^	0.00 × 10^+00^
F11									
AVG	0.00 × 10^+00^	0.00 × 10^+00^	0.00 × 10^+00^	0.00 × 10^+00^	0.00 × 10^+00^	0.00 × 10^+00^	0.00 × 10^+00^	0.00 × 10^+00^	0.00 × 10^+00^
STD	0.00 × 10^+00^	0.00 × 10^+00^	0.00 × 10^+00^	0.00 × 10^+00^	0.00 × 10^+00^	0.00 × 10^+00^	0.00 × 10^+00^	0.00 × 10^+00^	0.00 × 10^+00^
F12									
AVG	1.20 × 10^−02^	3.95 × 10^−02^	7.04 × 10^−02^	1.30 × 10^−02^	2.64 × 10^−02^	5.49 × 10^−02^	4.62 × 10^−03^	1.79 × 10^−02^	2.91 × 10^−02^
STD	9.09 × 10^−03^	2.16 × 10^−02^	4.94 × 10^−02^	9.74 × 10^−03^	1.64 × 10^−02^	4.07 × 10^−02^	4.61 × 10^−03^	1.84 × 10^−02^	2.11 × 10^−02^
F13									
AVG	6.63 × 10^−02^	4.03 × 10^−02^	4.77 × 10^−02^	7.70 × 10^−02^	2.11 × 10^−02^	6.56 × 10^−02^	6.36 × 10^−02^	6.53 × 10^−02^	8.61 × 10^−02^
STD	1.31 × 10^−01^	7.65 × 10^−02^	7.40 × 10^−02^	1.03 × 10^−01^	4.55 × 10^−02^	1.03 × 10^−01^	1.21 × 10^−01^	1.20 × 10^−01^	1.29 × 10^−01^
F14									
AVG	4.16 × 10^+00^	4.47 × 10^+00^	5.15 × 10^+00^	4.53 × 10^+00^	3.98 × 10^+00^	5.50 × 10^+00^	1.95 × 10^+00^	4.11 × 10^+00^	4.50 × 10^+00^
STD	4.17 × 10^+00^	3.50 × 10^+00^	4.03 × 10^+00^	3.88 × 10^+00^	3.01 × 10^+00^	4.13 × 10^+00^	2.07 × 10^+00^	3.92 × 10^+00^	3.84 × 10^+00^
F15									
AVG	5.12 × 10^−04^	4.90 × 10^−04^	4.79 × 10^−04^	5.58 × 10^−04^	4.91 × 10^−04^	5.03 × 10^−04^	5.16 × 10^−04^	4.68 × 10^−04^	4.26 × 10^−04^
STD	1.73 × 10^−04^	1.50 × 10^−04^	1.09 × 10^−04^	2.44 × 10^−04^	1.78 × 10^−04^	1.15 × 10^−04^	1.89 × 10^−04^	1.24 × 10^−04^	1.33 × 10^−04^
F16									
AVG	−1.03 × 10^+00^	−1.03 × 10^+00^	−1.03 × 10^+00^	−1.03 × 10^+00^	−1.03 × 10^+00^	−1.03 × 10^+00^	−1.03 × 10^+00^	−1.03 × 10^+00^	−1.03 × 10^+00^
STD	4.97 × 10^−16^	4.59 × 10^−16^	4.66 × 10^−16^	4.79 × 10^−16^	4.97 × 10^−16^	4.74 × 10^−16^	5.68 × 10^−16^	4.65 × 10^−16^	4.70 × 10^−16^
F17									
AVG	3.98 × 10^−01^	3.98 × 10^−01^	3.98 × 10^−01^	3.98 × 10^−01^	3.98 × 10^−01^	3.98 × 10^−01^	3.98 × 10^−01^	3.98 × 10^−01^	3.98 × 10^−01^
STD	0.00 × 10^+00^	7.71 × 10^−16^	5.42 × 10^−16^	3.24 × 10^−16^	0.00 × 10^+00^	3.24 × 10^−16^	0.00 × 10^+00^	0.00 × 10^+00^	9.73 × 10^−16^
F18									
AVG	3.00 × 10^+00^	3.00 × 10^+00^	3.00 × 10^+00^	3.00 × 10^+00^	3.00 × 10^+00^	3.00 × 10^+00^	3.00 × 10^+00^	3.00 × 10^+00^	3.00 × 10^+00^
STD	6.70 × 10^−04^	1.68 × 10^−06^	3.07 × 10^−04^	4.99 × 10^−10^	3.19 × 10^−08^	2.38 × 10^−05^	4.61 × 10^−11^	9.04 × 10^−10^	8.12 × 10^−06^
F19									
AVG	−3.86 × 10^+00^	−3.86 × 10^+00^	−3.86 × 10^+00^	−3.86 × 10^+00^	−3.86 × 10^+00^	−3.86 × 10^+00^	−3.86 × 10^+00^	−3.86 × 10^+00^	−3.86 × 10^+00^
STD	1.26 × 10^−11^	4.11 × 10^−06^	3.99 × 10^−04^	4.24 × 10^−12^	1.17 × 10^−10^	1.99 × 10^−06^	2.59 × 10^−13^	1.02 × 10^−09^	8.52 × 10^−06^
F20									
AVG	−3.32 × 10^+00^	−3.32 × 10^+00^	−3.32 × 10^+00^	−3.32 × 10^+00^	−3.32 × 10^+00^	−3.32 × 10^+00^	−3.32 × 10^+00^	−3.31 × 10^+00^	−3.32 × 10^+00^
STD	1.63 × 10^−10^	1.87 × 10^−04^	1.61 × 10^−08^	1.02 × 10^−10^	1.10 × 10^−08^	4.77 × 10^−08^	2.17 × 10^−02^	3.38 × 10^−02^	3.82 × 10^−04^
F21									
AVG	−9.14 × 10^+00^	−9.81 × 10^+00^	−1.02 × 10^+01^	−9.65 × 10^+00^	−1.02 × 10^+01^	−1.02 × 10^+01^	−9.98 × 10^+00^	−1.02 × 10^+01^	−1.02 × 10^+01^
STD	2.06 × 10^+00^	1.29 × 10^+00^	1.63 × 10^−09^	1.54 × 10^+00^	1.17 × 10^−12^	1.33 × 10^−11^	9.22 × 10^−01^	2.23 × 10^−10^	7.27 × 10^−11^
F22									
AVG	−9.35 × 10^+00^	−1.04 × 10^+01^	−1.02 × 10^+01^	−9.65 × 10^+00^	−1.04 × 10^+01^	−1.04 × 10^+01^	−1.04 × 10^+01^	−1.04 × 10^+01^	−1.04 × 10^+01^
STD	2.15 × 10^+00^	3.36 × 10^−11^	9.63 × 10^−01^	1.96 × 10^+00^	1.28 × 10^−11^	1.09 × 10^−10^	1.13 × 10^−06^	1.06 × 10^−09^	1.45 × 10^−07^
F23									
AVG	−8.88 × 10^+00^	−1.05 × 10^+01^	−1.05 × 10^+01^	−9.77 × 10^+00^	−1.05 × 10^+01^	−1.05 × 10^+01^	−1.03 × 10^+01^	−1.05 × 10^+01^	−1.05 × 10^+01^
STD	2.58 × 10^+00^	5.75 × 10^−10^	2.84 × 10^−09^	1.85 × 10^+00^	5.52 × 10^−11^	6.27 × 10^−10^	1.22 × 10^+00^	2.36 × 10^−06^	3.45 × 10^−02^
Friedman rank	5.7	5.0	5.2	5.4	4.0	4.7	5.2	4.8	5.1
Rank	9	4	6	8	1	2	7	3	5

**Table 7:** Comparison of benchmark function results.

Functions	Metric	Algorithms								
		ZOA	MIZOA	GWO	WOA	CSA	FA	SCA	AO	MZOA
F1	AVG	3.49 × 10^−249^	0.00 × 10^+00^	1.08 × 10^−27^	1.26 × 10^−71^	7.35 × 10^+02^	1.31 × 10^+01^	1.14 × 10^+01^	1.06 × 10^−109^	0.00 × 10^+00^
	STD	0.00 × 10^+00^	0.00 × 10^+00^	1.42 × 10^−27^	4.85 × 10^−71^	7.47 × 10^+02^	3.98 × 10^+00^	1.81 × 10^+01^	5.82 × 10^−109^	0.00 × 10^+00^
F2	AVG	1.76 × 10^−130^	0.00 × 10^+00^	1.01 × 10^−16^	1.09 × 10^−49^	7.27 × 10^+00^	1.17 × 10^+01^	1.47 × 10^−02^	1.63 × 10^−58^	0.00 × 10^+00^
	STD	4.75 × 10^−130^	0.00 × 10^+00^	5.40 × 10^−17^	5.31 × 10^−49^	5.81 × 10^+00^	1.39 × 10^+00^	1.41 × 10^−02^	8.94 × 10^−58^	0.00 × 10^+00^
F3	AVG	1.26 × 10^−154^	0.00 × 10^+00^	1.13 × 10^−05^	4.06 × 10^+04^	6.24 × 10^+03^	7.65 × 10^+02^	9.58 × 10^+03^	1.87 × 10^−108^	0.00 × 10^+00^
	STD	6.92 × 10^−154^	0.00 × 10^+00^	2.90 × 10^−05^	1.51 × 10^+04^	2.66 × 10^+03^	1.73 × 10^+03^	4.47 × 10^+03^	1.03 × 10^−107^	0.00 × 10^+00^
F4	AVG	2.05 × 10^−113^	0.00 × 10^+00^	8.61 × 10^−07^	4.38 × 10^+01^	6.47 × 10^+00^	3.46 × 10^+01^	3.51 × 10^+01^	1.76 × 10^−53^	0.00 × 10^+00^
	STD	6.85 × 10^−113^	0.00 × 10^+00^	1.00 × 10^−06^	3.05 × 10^+01^	5.06 × 10^+00^	8.17 × 10^+00^	1.25 × 10^+01^	9.65 × 10^−53^	0.00 × 10^+00^
F5	AVG	2.88 × 10^+01^	1.16 × 10^+00^	2.71 × 10^+01^	2.79 × 10^+01^	1.79 × 10^+04^	1.94 × 10^+04^	1.93 × 10^+04^	3.08 × 10^−03^	2.46 × 10^+01^
	STD	1.07 × 10^−01^	2.42 × 10^+00^	7.18 × 10^−01^	4.28 × 10^−01^	5.53 × 10^+04^	1.15 × 10^+04^	3.45 × 10^+04^	4.35 × 10^−03^	1.24 × 10^+00^
F6	AVG	0.00 × 10^+00^	0.00 × 10^+00^	0.00 × 10^+00^	0.00 × 10^+00^	5.47 × 10^+02^	2.30 × 10^+01^	2.31 × 10^+01^	0.00 × 10^+00^	8.57 × 10^−02^
	STD	0.00 × 10^+00^	0.00 × 10^+00^	0.00 × 10^+00^	0.00 × 10^+00^	5.91 × 10^+02^	1.34 × 10^+01^	6.07 × 10^+01^	0.00 × 10^+00^	1.55 × 10^−01^
F7	AVG	1.19 × 10^−04^	5.59 × 10^−05^	2.01 × 10^−03^	4.52 × 10^−03^	6.47 × 10^−01^	1.43 × 10^+01^	1.25 × 10^−01^	1.30 × 10^−04^	1.76 × 10^−05^
	STD	1.08 × 10^−04^	5.98 × 10^−05^	8.32 × 10^−04^	4.91 × 10^−03^	2.83 × 10^−01^	5.31 × 10^+00^	1.83 × 10^−01^	1.08 × 10^−04^	1.85 × 10^−05^
F8	AVG	−5.89 × 10^+03^	−1.12 × 10^+04^	−6.06 × 10^+03^	−1.08 × 10^+04^	−1.06 × 10^+04^	−8.51 × 10^+03^	−3.65 × 10^+03^	−8.71 × 10^+03^	−9.49 × 10^+03^
	STD	7.40 × 10^+02^	1.30 × 10^+03^	1.21 × 10^+03^	1.90 × 10^+03^	1.63 × 10^+03^	7.54 × 10^+02^	2.38 × 10^+02^	3.62 × 10^+03^	8.19 × 10^+02^
F9	AVG	0.00 × 10^+00^	0.00 × 10^+00^	3.01 × 10^+00^	1.89 × 10^−15^	3.88 × 10^+01^	2.16 × 10^+02^	3.47 × 10^+01^	0.00 × 10^+00^	0.00 × 10^+00^
	STD	0.00 × 10^+00^	0.00 × 10^+00^	3.81 × 10^+00^	1.04 × 10^−14^	5.23 × 10^+01^	1.85 × 10^+01^	2.95 × 10^+01^	0.00 × 10^+00^	0.00 × 10^+00^
F10	AVG	4.44 × 10^−16^	4.44 × 10^−16^	1.02 × 10^−13^	3.88 × 10^−15^	3.63 × 10^−01^	4.35 × 10^+00^	1.44 × 10^+01^	4.44 × 10^−16^	8.88 × 10^−16^
	STD	0.00 × 10^+00^	0.00 × 10^+00^	1.56 × 10^−14^	2.55 × 10^−15^	3.34 × 10^−01^	6.78 × 10^−01^	8.68 × 10^+00^	0.00 × 10^+00^	0.00 × 10^+00^
F11	AVG	0.00 × 10^+00^	0.00 × 10^+00^	4.63 × 10^−03^	9.94 × 10^−05^	2.45 × 10^+01^	5.23 × 10^−01^	8.86 × 10^−01^	0.00 × 10^+00^	0.00 × 10^+00^
	STD	0.00 × 10^+00^	0.00 × 10^+00^	9.66 × 10^−03^	3.89 × 10^−02^	1.60 × 10^+01^	1.18 × 10^−01^	3.58 × 10^−01^	0.00 × 10^+00^	0.00 × 10^+00^
F12	AVG	4.19 × 10^−01^	3.39 × 10^−02^	4.61 × 10^−02^	2.14 × 10^−02^	3.31 × 10^+00^	1.59 × 10^+02^	2.19 × 10^+04^	3.81 × 10^−06^	2.79 × 10^−03^
	STD	1.41 × 10^−01^	1.73 × 10^−02^	2.17 × 10^−02^	1.12 × 10^−02^	5.41 × 10^+00^	4.09 × 10^+02^	6.40 × 10^+04^	6.21 × 10^−06^	4.05 × 10^−03^
F13	AVG	2.39 × 10^+00^	2.04 × 10^−02^	6.33 × 10^−01^	4.83 × 10^−01^	9.08 × 10^+02^	3.17 × 10^+02^	1.07 × 10^+05^	1.51 × 10^−05^	6.70 × 10^−02^
	STD	2.86 × 10^−01^	4.22 × 10^−02^	2.24 × 10^−01^	2.71 × 10^−01^	2.81 × 10^+03^	5.60 × 10^+02^	2.78 × 10^+05^	2.00 × 10^−05^	8.87 × 10^−02^
F14	AVG	4.27 × 10^+00^	4.34 × 10^+00^	5.14 × 10^+00^	2.96 × 10^+00^	1.13 × 10^+00^	1.06 × 10^+00^	1.99 × 10^+00^	3.77 × 10^+00^	1.52 × 10^+00^
	STD	3.36 × 10^+00^	3.49 × 10^+00^	4.55 × 10^+00^	3.37 × 10^+00^	3.44 × 10^−01^	2.52 × 10^−01^	1.91 × 10^+00^	4.45 × 10^+00^	1.85 × 10^+00^
F15	AVG	1.71 × 10^−03^	4.69 × 10^−04^	3.07 × 10^−03^	7.57 × 10^−03^	2.11 × 10^−03^	1.08 × 10^−03^	1.20 × 10^−03^	5.09 × 10^−04^	3.17 × 10^−04^
	STD	5.09 × 10^−03^	1.58 × 10^−04^	6.90 × 10^−03^	4.77 × 10^−04^	3.17 × 10^−03^	3.40 × 10^−04^	3.31 × 10^−04^	9.53 × 10^−05^	1.90 × 10^−05^
F16	AVG	−1.03 × 10^+00^	−1.03 × 10^+00^	−1.03 × 10^+00^	−1.03 × 10^+00^	−1.03 × 10^+00^	−1.03 × 10^+00^	−1.03 × 10^+00^	−1.03 × 10^+00^	−1.03 × 10^+00^
	STD	1.62 × 10^−11^	4.88 × 10^−16^	3.16 × 10^−08^	1.39 × 10^−09^	3.26 × 10^−11^	1.24 × 10^−04^	3.86 × 10^−05^	5.90 × 10^−04^	1.14 × 10^−11^
F17	AVG	3.98 × 10^−01^	3.98 × 10^−01^	3.98 × 10^−01^	3.98 × 10^−01^	3.98 × 10^−01^	3.98 × 10^−01^	4.00 × 10^−01^	3.98 × 10^−01^	3.98 × 10^−01^
	STD	9.69 × 10^−10^	0.00 × 10^+00^	7.72 × 10^−05^	3.15 × 10^−05^	6.12 × 10^−14^	9.73 × 10^−05^	2.10 × 10^−03^	1.87 × 10^−04^	1.18 × 10^−09^
F18	AVG	3.80 × 10^+00^	3.00 × 10^+00^	3.10 × 10^+00^	3.00 × 10^+00^	3.03 × 10^+00^	3.00 × 10^+00^	3.00 × 10^+00^	3.03 × 10^+00^	3.00 × 10^+00^
	STD	4.85 × 10^+00^	1.01 × 10^−07^	2.06 × 10^−04^	5.32 × 10^−05^	1.58 × 10^−04^	6.38 × 10^−03^	2.12 × 10^−04^	2.59 × 10^−02^	5.37 × 10^−08^
F19	AVG	−3.86 × 10^+00^	−3.86 × 10^+00^	−3.86 × 10^+00^	−3.85 × 10^+00^	−3.83 × 10^+00^	−3.85 × 10^+00^	−3.85 × 10^+00^	−3.86 × 10^+00^	−3.86 × 10^+00^
	STD	7.18 × 10^−04^	8.01 × 10^−10^	2.83 × 10^−03^	1.16 × 10^−02^	3.42 × 10^−02^	1.59 × 10^−02^	3.07 × 10^−03^	4.70 × 10^−03^	2.00 × 10^−03^
F20	AVG	−3.29 × 10^+00^	−3.32 × 10^+00^	−3.25 × 10^+00^	−3.17 × 10^+00^	−3.13 × 10^+00^	−2.98 × 10^+00^	−2.94 × 10^+00^	−3.18 × 10^+00^	−3.28 × 10^+00^
	STD	5.82 × 10^−02^	3.44 × 10^−09^	9.55 × 10^−02^	1.72 × 10^−01^	1.27 × 10^−01^	2.40 × 10^−01^	3.02 × 10^−01^	9.20 × 10^−02^	9.65 × 10^−02^
F21	AVG	−9.47 × 10^+00^	−1.02 × 10^+01^	−8.82 × 10^+00^	−8.10 × 10^+00^	−1.01 × 10^+01^	−8.04 × 10^+00^	−2.49 × 10^+00^	−1.01 × 10^+01^	−8.99 × 10^+00^
	STD	1.76 × 10^+00^	1.29 × 10^−12^	2.76 × 10^+00^	2.53 × 10^+00^	5.13 × 10^−02^	2.47 × 10^+00^	1.89 × 10^+00^	2.82 × 10^−02^	2.68 × 10^+00^
F22	AVG	−9.30 × 10^+00^	−1.04 × 10^+01^	−1.02 × 10^+01^	−7.65 × 10^+00^	−7.69 × 10^+00^	−9.28 × 10^+00^	−3.75 × 10^+00^	−1.04 × 10^+01^	−9.05 × 10^+00^
	STD	2.25 × 10^+00^	3.17 × 10^−11^	9.70 × 10^−01^	3.07 × 10^+00^	3.45 × 10^+00^	1.85 × 10^+00^	1.91 × 10^+00^	4.53 × 10^−02^	2.78 × 10^+00^
F23	AVG	−9.45 × 10^+00^	−1.05 × 10^+01^	−1.04 × 10^+01^	−7.62 × 10^+00^	−1.05 × 10^+01^	−8.73 × 10^+00^	−3.93 × 10^+00^	−1.05 × 10^+01^	−8.43 × 10^+00^
	STD	2.24 × 10^+00^	8.01 × 10^−09^	9.79 × 10^−01^	3.27 × 10^+00^	1.27 × 10^−01^	2.86 × 10^+00^	1.53 × 10^+00^	2.11 × 10^−02^	3.31 × 10^+00^
Friedman rank		4.1	2.2	5.4	5.6	6.3	6.6	7.2	3.8	3.4
Rank		4	1	5	6	7	8	9	3	2

**Table 8:** Friedman test results.

*p*-Value	ZOA	MIZOA	GWO	WOA	CSA	FA	SCA	AO	MZOA
1.12 × 10^−26^	4.1	2.2	5.4	5.6	6.3	6.6	7.2	3.8	3.4

**Table 9:** *p*-value of the Wilcoxon test.

Functions	Algorithms						
	ZOA vs. MIZOA	MIZOA vs. GWO	MIZOA vs. WOA	MIZOA vs. CSA	MIZOA vs. FA	MIZOA vs. SCA	MIZOA vs. AO
F1	1.21 × 10^−12^	1.21 × 10^−12^	1.21 × 10^−12^	1.21 × 10^−12^	1.21 × 10^−12^	1.21 × 10^−12^	1.21 × 10^−12^
F2	1.21 × 10^−12^	1.21 × 10^−12^	1.21 × 10^−12^	1.21 × 10^−12^	1.21 × 10^−12^	1.21 × 10^−12^	1.21 × 10^−12^
F3	1.21 × 10^−12^	1.21 × 10^−12^	1.21 × 10^−12^	1.21 × 10^−12^	1.21 × 10^−12^	1.21 × 10^−12^	1.21 × 10^−12^
F4	1.21 × 10^−12^	1.21 × 10^−12^	1.21 × 10^−12^	1.21 × 10^−12^	1.21 × 10^−12^	1.21 × 10^−12^	1.21 × 10^−12^
F5	3.02 × 10^−11^	3.02 × 10^−11^	3.02 × 10^−11^	3.02 × 10^−11^	3.02 × 10^−11^	3.02 × 10^−11^	3.02 × 10^−11^
F6	1.00 × 10^+00^	1.00 × 10^+00^	1.00 × 10^+00^	1.21 × 10^−12^	1.19 × 10^−12^	1.91 × 10^−09^	1.00 × 10^+00^
F7	5.32 × 10^−03^	3.02 × 10^−11^	4.08 × 10^−11^	3.02 × 10^−11^	3.02 × 10^−11^	3.02 × 10^−11^	2.06 × 10^−01^
F8	3.02 × 10^−11^	3.02 × 10^−11^	1.38 × 10^−02^	9.05 × 10^−02^	2.19 × 10^−08^	3.02 × 10^−11^	2.71 × 10^−02^
F9	1.00 × 10^+00^	4.49 × 10^−12^	3.34 × 10^−01^	1.21 × 10^−12^	1.21 × 10^−12^	1.21 × 10^−12^	1.00 × 10^+00^
F10	1.00 × 10^+00^	1.14 × 10^−12^	2.85 × 10^−07^	1.21 × 10^−12^	1.21 × 10^−12^	1.21 × 10^−12^	1.00 × 10^+00^
F11	1.00 × 10^+00^	2.16 × 10^−02^	3.34 × 10^−01^	1.21 × 10^−12^	1.21 × 10^−12^	1.21 × 10^−12^	1.00 × 10^+00^
F12	3.02 × 10^−11^	6.77 × 10^−05^	7.17 × 10^−01^	3.16 × 10^−10^	3.02 × 10^−11^	3.02 × 10^−11^	3.02 × 10^−11^
F13	3.02 × 10^−11^	8.15 × 10^−11^	4.62 × 10^−10^	3.02 × 10^−11^	3.02 × 10^−11^	3.02 × 10^−11^	3.02 × 10^−11^
F14	2.89 × 10^−01^	5.94 × 10^−01^	4.84 × 10^−02^	2.65 × 10^−06^	1.24 × 10^−04^	1.32 × 10^−02^	2.84 × 10^−01^
F15	5.27 × 10^−05^	2.97 × 10^−01^	6.73 × 10^−01^	1.73 × 10^−07^	1.03 × 10^−06^	1.53 × 10^−05^	2.06 × 10^−01^
F16	4.78 × 10^−08^	1.21 × 10^−12^	1.21 × 10^−12^	1.37 × 10^−03^	1.21 × 10^−12^	1.21 × 10^−12^	1.21 × 10^−12^
F17	1.95 × 10^−09^	1.21 × 10^−12^	1.21 × 10^−12^	1.61 × 10^−01^	1.21 × 10^−12^	1.21 × 10^−12^	1.21 × 10^−12^
F18	2.66 × 10^−03^	8.01 × 10^−10^	8.80 × 10^−10^	1.06 × 10^−02^	4.03 × 10^−11^	1.31 × 10^−10^	2.98 × 10^−11^
F19	1.10 × 10^−11^	1.10 × 10^−11^	1.10 × 10^−11^	1.10 × 10^−11^	1.10 × 10^−11^	1.10 × 10^−11^	1.10 × 10^−11^
F20	4.08 × 10^−11^	3.02 × 10^−11^	3.02 × 10^−11^	3.02 × 10^−11^	3.02 × 10^−11^	3.02 × 10^−11^	3.02 × 10^−11^
F21	1.21 × 10^−12^	1.21 × 10^−12^	1.21 × 10^−12^	1.21 × 10^−12^	1.21 × 10^−12^	1.21 × 10^−12^	1.21 × 10^−12^
F22	7.76 × 10^−12^	7.76 × 10^−12^	7.76 × 10^−12^	7.76 × 10^−12^	7.76 × 10^−12^	7.76 × 10^−12^	7.76 × 10^−12^
F23	6.46 × 10^−12^	6.46 × 10^−12^	6.46 × 10^−12^	6.46 × 10^−12^	6.46 × 10^−12^	6.46 × 10^−12^	6.46 × 10^−12^
+/−/=	18/1/4	20/2/1	18/4/1	21/2/0	23/0/0	23/0/0	16/3/4

**Table 10:** Performance Comparison of Algorithms on Tension/Compression Spring Design.

Algorithms	Avg	Std	Best	d	D	n
ZOA	1.348 × 10^−02^	6.715 × 10^−04^	1.281 × 10^−02^	5.336 × 10^−02^	3.984 × 10^−01^	9.208 × 10^+00^
MIZOA	1.271 × 10^−02^	6.333 × 10^−05^	1.267 × 10^−02^	5.102 × 10^−02^	3.409 × 10^−01^	1.228 × 10^+01^
GWO	1.284 × 10^−02^	1.980 × 10^−04^	1.267 × 10^−02^	5.000 × 10^−02^	3.174 × 10^−01^	1.404 × 10^+01^
WOA	1.435 × 10^−02^	1.556 × 10^−03^	1.267 × 10^−02^	5.454 × 10^−02^	4.292 × 10^−01^	8.031 × 10^+00^
CSA	1.296 × 10^−02^	3.176 × 10^−04^	1.267 × 10^−02^	5.160 × 10^−02^	3.546 × 10^−01^	1.141 × 10^+01^
FA	2.958 × 10^+100^	3.729 × 10^+100^	1.904 × 10^−02^	6.690 × 10^−02^	7.587 × 10^−01^	6.174 × 10^+00^
SCA	1.314 × 10^−02^	1.434 × 10^−04^	1.277 × 10^−02^	5.000 × 10^−02^	3.174 × 10^−01^	1.443 × 10^+01^
AO	1.874 × 10^−02^	3.280 × 10^−03^	1.352 × 10^−02^	5.644 × 10^−02^	4.692 × 10^−01^	8.646 × 10^+00^

**Table 11:** Statistical Comparison of Algorithms on the Speed Reducer Design Problem.

Algorithms	Avg	Std	Best	Worst
ZOA	3.016 × 10^+03^	7.313 × 10^+01^	2.997 × 10^+03^	3.400 × 10^+03^
MIZOA	2.995 × 10^+03^	8.304 × 10^−01^	2.994 × 10^+03^	2.998 × 10^+03^
GWO	3.013 × 10^+03^	6.616 × 10^+00^	3.003 × 10^+03^	3.028 × 10^+03^
WOA	3.485 × 10^+03^	6.620 × 10^+02^	3.018 × 10^+03^	5.382 × 10^+03^
CSA	4.867 × 10^+97^	1.731 × 10^+98^	3.088 × 10^+03^	9.268 × 10^+98^
FA	4.394 × 10^+97^	7.760 × 10^+97^	3.498 × 10^+03^	2.649 × 10^+98^
SCA	3.143 × 10^+03^	4.653 × 10^+01^	3.053 × 10^+03^	3.230 × 10^+03^
AO	3.377 × 10^+03^	4.839 × 10^+02^	3.070 × 10^+03^	4.636 × 10^+03^

**Table 12:** Optimal Design Variables for the Speed Reducer from Different Algorithms.

Algorithms	Optimum Variables						Optimum Cost
	m	z	L1	L2	D1	D2	
ZOA	0.7	17	7.594419272	7.729178376	3.35079056	5.28669791	2.997 × 10^+03^
MIZOA	0.7	17	7.3	7.715322526	3.350216563	5.286654495	2.994 × 10^+03^
GWO	0.700205191	17	7.652455574	7.782475322	3.353167341	5.288761303	3.003 × 10^+03^
WOA	0.7	17	7.3	8.141941553	3.350214734	5.288620974	3.018 × 10^+03^
CSA	0.7	17	7.960030823	7.750113448	3.589731062	5.318284935	3.088 × 10^+03^
FA	0.717631318	18.36144647	7.700559378	8.266662603	3.55627816	5.364287331	3.498 × 10^+03^
SCA	0.7	17	7.3	8.3	3.372006288	5.287260513	3.053 × 10^+03^
AO	0.7	17	7.657689296	8.278979684	3.391896062	5.302402366	3.070 × 10^+03^

**Table 13:** Statistical Comparison of Algorithms on the Pressure Vessel Design Problem.

Algorithms	Avg	Std	Best	Worst
ZOA	1.723 × 10^+04^	1.566 × 10^+04^	6.840 × 10^+03^	7.829 × 10^+04^
MIZOA	5.983 × 10^+03^	1.406 × 10^+02^	5.886 × 10^+03^	6.669 × 10^+03^
GWO	6.213 × 10^+03^	4.159 × 10^+02^	5.895 × 10^+03^	7.317 × 10^+03^
WOA	1.966 × 10^+04^	5.073 × 10^+04^	6.704 × 10^+03^	4.452 × 10^+04^
CSA	9.436 × 10^+03^	1.219 × 10^+04^	6.127 × 10^+03^	4.372 × 10^+04^
FA	2.314 × 10^+05^	1.524 × 10^+05^	7.363 × 10^+04^	4.677 × 10^+05^
SCA	7.422 × 10^+03^	6.839 × 10^+02^	6.379 × 10^+03^	9.152 × 10^+03^
AO	7.030 × 10^+03^	4.807 × 10^+02^	6.176 × 10^+03^	8.140 × 10^+03^

**Table 14:** Optimal variable values for the pressure vessel design problem obtained by different algorithms.

Algorithms	Optimum Variables				Optimum Cost
	T2	T1	R	L	
ZOA	1.08666	0.57091	56.30222	55.06889	6.840 × 10^+03^
MIZOA	0.77841	0.38477	40.33233	199.82319	5.886 × 10^+03^
GWO	0.77914	0.38712	40.35431	199.52196	5.895 × 10^+03^
WOA	0.87863	0.60251	45.48166	138.78411	6.704 × 10^+03^
CSA	0.89590	0.44303	46.41959	129.81384	6.127 × 10^+03^
FA	4.13418	3.91925	54.03215	181.01543	7.363 × 10^+04^
SCA	0.81474	0.40607	41.27055	200.00000	6.379 × 10^+03^
AO	0.81710	0.46449	42.15069	176.21460	6.176 × 10^+03^

**Table 15:** Performance Comparison of Path Planning Algorithms.

Grid Map	Algorithms	Best	Avg	Std
20 × 20	ZOA	28.5417	33.0766	1.7938
	MIZOA	27.2472	27.8446	0.3962
	MZOA	27.6381	30.2604	1.1259
	WOA	28.1750	29.1750	1.8167
	CSA	35.2401	35.3591	0.1187
	SCA	30.0591	30.7452	0.4519
	AO	27.6025	28.5018	1.0182
30 × 30	ZOA	45.0010	51.4118	2.5078
	MIZOA	41.8407	43.0308	0.6261
	MZOA	42.8052	46.8516	1.9075
	WOA	42.2931	45.8817	2.7704
	CSA	51.9923	53.1474	0.3678
	SCA	46.9424	49.2072	4.2251
	AO	42.9833	45.2934	2.0375

## Data Availability

The raw data supporting the conclusions of this article will be available from the authors on request.
